# TRIB2 modulates proteasome function to reduce ubiquitin stability and protect liver cancer cells against oxidative stress

**DOI:** 10.1038/s41419-020-03299-8

**Published:** 2021-01-07

**Authors:** Susu Guo, Yuxin Chen, Yueyue Yang, Xiao Zhang, Lifang Ma, Xiangfei Xue, Yongxia Qiao, Jiayi Wang

**Affiliations:** 1 Department of Clinical Laboratory, Shanghai Tenth People’s Hospital of Tongji University, Shanghai 200072, China; 2 West China Second University Hospital, Sichuan University, Sichuan 610041, China; 3 Shanghai Institute of Thoracic Oncology, Shanghai Chest Hospital, Shanghai Jiao Tong University, Shanghai 200030, China; 4 School of Public Health, Shanghai Jiao Tong University School of Medicine, Shanghai 200025, China

**Keywords:** Oncogenes, Cancer

## Abstract

The regulation of homeostasis in the Ubiquitin (Ub) proteasome system (UPS) is likely to be important for the development of liver cancer. Tribbles homolog 2 (TRIB2) is known to affect Ub E3 ligases (E3s) in liver cancer. However, whether TRIB2 regulates the UPS in other ways and the relevant mechanisms are still unknown. Here, we reveal that TRIB2 decreased Ub levels largely by stimulating proteasome degradation of Ub. In the proteasome, proteasome 20S subunit beta 5 (PSMB5) was critical for the function of TRIB2, although it did not directly interact with TRIB2. However, poly (rC) binding protein 2 (PCBP2), which was identified by mass spectrometry, directly interacted with both TRIB2 and PSMB5. PCBP2 was a prerequisite for the TRIB2 induction of PSMB5 activity and decreased Ub levels. A significant correlation between TRIB2 and PCBP2 was revealed in liver cancer specimens. Interestingly, TRIB2 suppressed the K48-ubiquitination of PCBP2 to increase its level. Therefore, a model showing that TRIB2 cooperates and stimulates PCBP2 to reduce Ub levels was established. Additionally, the reduction in Ub levels induced by TRIB2 and PCBP2 was dependent on K48-ubiquitination. PCBP2 was one of the possible downstream factors of TRIB2 and their interaction relied on the DQLVPD element of TRIB2 and the KH3 domain of PCBP2. This interaction was necessary to maintain the viability of the liver cancer cells and promote tumor growth. Mechanistically, glutathione peroxidase 4 functioned as one of the terminal effectors of TRIB2 and PCBP2 to protect liver cancer cells from oxidative damage. Taken together, the data indicate that, in addition to affecting E3s, TRIB2 plays a critical role in regulating UPS by modulating PSMB5 activity in proteasome to reduce Ub flux, and that targeting TRIB2 might be helpful in liver cancer treatments by enhancing the oxidative damage induced by therapeutic agents.

## Introduction

Lack of understanding of the molecular mechanism underlying liver tumorigenesis has restricted the development of treatment. TRIB2 is a pseudokinase shown to be a potent oncogene in a variety of malignancies^1^. The *Trib2* gene was first identified as a murine myeloid oncogene^2^. However, the role of TRIB2 in human liver cancer cells is not completely known.

A major carcinogenic mechanism of TRIB2 involves substrate degradation and protein stability^3^. TRIB2 acts as a scaffold protein recruiting E3s to facilitate the ubiquitination and degradation of substrates via the UPS in leukemia and lung cancer^4,5^. In our previous works, we noticed that TRIB2 exerts a similar function in liver cancer cells^6–8^. Therefore, we and others have proposed a function of TRIB2 as a molecular adapter linking E3s to the modulation of the UPS^4,6^. However, whether TRIB2 alternately functions to regulate the UPS, especially the regulation of Ub level, and the mechanisms involved remain unclear.

Ub levels depend on cell conditions^9^. It is initially expressed in the form of various precursors that are transcribed from four *Ub* host genes, i.e., *UBA52*, *UBA80*, *UBB*, and *UBC*. In the presence of deubiquitinating enzymes (DUBs), Ub precursors are cleaved into units of monomeric Ub (mono Ub), which can be conjugated to substrates in a process facilitated by ubiquitin-activating enzyme (E1), ubiquitin-conjugating enzyme (E2) and E3s^7^. Although a stable protein, Ub can be degraded under certain conditions. Ub can be polyubiquitinated by K48-linked chains before targeting to the proteasome^10^. Ub can also be degraded simultaneously with its conjugated substrates^11^. Nevertheless, the proteasome is the ultimate mediator of Ub degradation. Interestingly, DUBs have alternative functions, such as recycling the Ub released from substrates, thus preventing excessive Ub degradation^12^. To date, most studies have focused on the degradation mechanism of specific substrates; however, few studies have concentrated on Ub itself.

The UPS participates in various biological processes^13–15^. Its role in protecting against oxidative stress (OS) is one of its most important functions^16^. Cell damage is caused by OS due to excessive reactive oxygen species (ROS) and their derivatives, such as superoxide and hydrogen peroxide. A functional UPS is required to cope with various types of OS because it selectively degrades oxidatively damaged proteins^17^. In addition, antioxidant enzymes, such as glutathione peroxidase 4 (GPX4), act in concert to remove various ROS^18^. Some evidence shows that TRIB2 protects cells against OS^19^. Notably, PCBP2, one of the major cellular poly (rC)-binding proteins, not only inhibits ROS production^20^ but also regulates substrate ubiquitination^21^. In addition, PCBP2 was found to be closely related to the development and progression of some types of cancers^22,23^. However, whether TRIB2 interacts with PCBP2 to regulate Ub levels and how this interaction influences the viability of liver cancer cells under OS remain unclear.

Therefore, we investigated whether TRIB2 modulates the UPS by regulating Ub levels independent of E3s and the possible mechanisms involved. The interaction and function between TRIB2 and PCBP2 was also investigated in liver cancer cells. Our data provide new evidence that further supports the supposition that the UPS is tightly regulated by TRIB2, and that blocking its function might be helpful in the treatment of liver cancer by increasing oxidative damage.

## Materials and methods

### Mouse experiments and tissue samples

A total of 45 female athymic nude mouse (5–6 weeks old and weighing 20–25 g) were randomly divided into groups and subcutaneously injected in the back with 1 × 10^7^ Bel-7402 cells. Tumors were measured three times weekly, and the tumor volume was calculated as follows: length × width^2^/2. All animal experiments were conducted with the approval of the Institutional Review Board of the Shanghai Tenth People’s Hospital. The slices of the tissue microarray assay (TMA, lv20811) were purchased from US Biomax through the agent Alenabio (Xi’an, China).

### Cell culture (3D culture) and vectors

HL-7702, Bel-7402, Bel-7404, SMMC-7721, HepG2, and Huh7 cell lines were get from our previous study^24^. HEK293T and A549 cell lines were purchased from Zorin (Shanghai, China). Authentication of these cell lines was performed by short tandem repeat (STR) markers, and no mycoplasma contamination was detected. Cells were cultured in DMEM (SH30243, HyClone, Logan, UT, USA) supplemented with 10% fetal bovine serum (Sagecreation, Beijing, China) and a 1% penicillin-streptomycin solution (15140122, Gibco, Grand Island, NY, USA). The cells were treated with CHX (50 μg/ml, HY-12320, MCE, Shanghai, China), MG132 (8 or 50 µM, HY-13259, MCE), bortezomib (BTZ, 100 nM, HY-10227, MCE), EOAI3402143 (EOAI, 600 nM, HY-111408, MCE), VLX1570 (20 µM, HY-12471, MCE), 3-methyladenine (3-MA, 5 mM, HY-19312, MCE), chloroquine diphosphate (CQ, 20 µM, HY-135811A, MCE), Z-Asp-CH2-DCB (ZACD, 100 µM, HY-113953, MCE), N-alpha-Tosyl-l-lysine chloromethyl ketone (TLCK, 50 μM, HY-112716, MCE), b-AP15 (5 μM, SG0020, Beyotime, Shanghai, China), doxycycline (Dox, 700 ng/ml, s4163, Selleck, TX, USA), RAS-selective lethal 3 (RSL3, 5 µM, S8155, Selleck), *t*-butyl hydroperoxide (*t*-BuOOH, 19997, Sigma, St. Louis, MO, USA), diquat (PS-365, Chem Service, West Chester, PA, USA), desferrioxamine (DFO, 25 µM, D9533, Sigma), and Afatinib (10 µM, HY-10261, MCE).

3D cultures were generated by using Cultrex BME (3432–005–01, Trevigen, Gaithersburg, MD, USA). Briefly, BME was spread over a 96-well plate. Cell culture medium was added at 1000/well atop the surface matrix. Cell growth was observed and recorded daily.

TRIB2-FLAG-expressing, TRIB2 shRNA (sh1 and sh2)-expressing, PCBP2-HA-expressing, Ub-HA-expressing and PCBP2 shRNA (sh1 and sh2)-expressing plasmids were generated as described in our previous studies^6,25,26^. UCH37 shRNA (TRCN0000007427) and USP14 shRNA (TRCN0000004128) were purchased from Dharmacon (lafayette, FL, USA). The PSMB5-HA-expressing, GPX4-expressing and GFP-expressing plasmids were purchased from Yuanjian Biotechnology (Shanghai, China). PARP1 shRNA-expressing, TRIB1-FLAG-expressing, and TRIB3-FLAG-expressing plasmids were purchased from Biolink (Shanghai, China). The PSMB5 sgRNA (117073), Cas9 (78166), PCBP1 (98362), PCBP3 (53957), K48-Ub-HA (17605), K63-Ub-HA (17606), and ATG5 sgRNA (99573) were purchased from Addgene (Watertown, MA, USA). The luciferase reporter plasmids for testing HIF1α activity were gifts from Prof. Xuyun Zhao (Shanghai Jiaotong University School of Medicine, Shanghai, China). The siRNAs targeting ATG7, POMP, MYPT1, and GRP78 were purchased from GenePharma (Shanghai, China) with the sequence listed in Supplementary Table S1. The sgRNAs targeting TRIB2 and PCBP2, shRNA targeting GPX4 (sh1 and sh2) and mutants of TRIB2 and PCBP2 were constructed by PCR with the primers listed in Supplementary Table S2.

### Immunoblotting (IB) and immunohistochemistry (IHC)

Conventional protocols were used for IB and IHC, with the details available elsewhere^25^. The following primary antibodies were used for IB: anti-TRIB1 (ab137717, 1:1000), anti-TRIB2 (ab117981, 1:1000), anti-TRIB3 (ab75846, 1:10,000), anti-PCBP1 (ab168377, 1:1000), anti-PCBP2 (ab184962, 1:1000), anti-PCBP3 (ab154252, 1:1000), anti-USP14 (ab192618, 1:5000), anti-UCH37 (ab133508, 1:10,000), anti-ATG7 (ab52472, 1:1000), anti-GPX4 (ab125066 and ab16739, 1:1000), anti-Calnexin (ab92573, 1:1000), anti-ATG5 (ab108327, 1:5000), anti-GRP78 (ab121390, 1:1000), anti-PSMB3 (ab230024, 1:1000), anti-PSMB5 (ab167341, 1:1000), anti-CREB (ab32096, 1:1000) and anti-PARP1 (ab32138, 1:1000), all purchased from Abcam (Cambridge, UK); anti-Ub (3936 or 3933, 1:1000), anti-GAPDH (5174, 1:2000), anti-HA (3724 or 2367, 1:1000), anti-FLAG (14793 or 8146, 1:1000), anti-K48-Ub (8081, 1:1000), anti-K63-Ub (5621, 1:1000), anti-LAMP2 (49067, 1:1000), anti-βTrCP (11984, 1:1000), anti-POMP (15141, 1:1000), anti-HIF1α (36169, 1:1000), anti-HIF1α-OH (3434, 1:1000), and anti-MYPT1 (8574, 1:1000), all purchased from Cell Signaling Technology (CST, Boston, MA, USA); anti-GFP (AG281, 1:1000) was purchased from Beyotime (Shanghai, China); anti-V5 (YM3005, 1:1000) and anti-PCBP3 (YN3731, 1:1000) were purchased from Immunoway (Plano, TX, USA); anti-PCBP2 (H00005094-M08) was purchased from abnova; and anti-PSMB5 (DF6728, 1:1000) was purchased from Affinity (Changzhou, Jiangsu Province, China). The nuclear extracts were prepared by a kit from Active motif (Carlsbad, CA, USA). The membranes were incubated with HRP-conjugated secondary antibodies (7076 and 7054, 1:2000, CST) and visualized using Clarity Western ECL substrate (1705060, Bio-Rad, Hercules, CA, USA). The antibodies used for IHC were anti-TRIB2 (ab117981, 1:50, Abcam) and anti-PCBP2 (ab184962, 1:50, Abcam). Signal detection was accomplished using IHC detection reagent (8114 or 8125, CST). The specimens were scored semiquantitatively on the basis of a well-established immunoreactivity score system (IRS). The IRS score was calculated by multiplying the score for the percentage of positive cells (4, >80%; 3, 51–80%; 2, 10–50%; 1, <10%; 0, 0%) with the score for the staining intensity (3, strong; 2, moderate; 1, mild; and 0, no staining), which results in IRS scores between 0 and 12.

### Quantitative RT-PCR (qPCR)

Total RNA was extracted by TRIzol reagent (15596018, Invitrogen, Carlsbad CA, USA). Then, the RNA was reverse transcribed into cDNA using a kit from Vazyme (R323–01, Nanjing, Jiangsu province, China). All reactions were carried out using SYBR Green mix (Q711–02, Vazyme). The data from the qRT-PCRs were analyzed by the Δ*C*_t_ method: Δ*C*_t_ = *C*_t (target gene)_ − *C*_t (GAPDH)_, ΔΔ*C*_t_ = Δ*C*_t (experiment group)_ − Δ*C*_t (control group)_. The relative expression level for a target gene in the stimulated cells was calculated as follows: relative mRNA level = 2^−ΔΔCt^. The primer sequences are available in Supplementary Table S3.

### Coimmunoprecipitation (co-IP)

Conventional co-IP was performed. Specifically, in vitro co-IP was performed according to the prior study^27^. Briefly, 1 µg purified protein was mixed with 5 µg antibodies in binding buffer (25 mM Tris-HCl, pH 7.2, 150 mM NaCl, 5 mM MgCl_2_, 1% NP-40, and 20 mM imidazole) to a final volume of 400 µl in an end-over-end rotator at room temperature for 1 h. Afterwards, 25 µl Pierce protein A/G magnetic beads (88803, Thermo Scientific, Waltham, MA, USA) were added to the antigen/antibody mixture, and incubated at room temperature for 1 h with mixing. Beads were subsequently collected and washed once with binding buffer to remove unbound proteins. After further incubation with 1 µg another purified proteins at 4 °C overnight in 400 µl of binding buffer, the beads were collected and washed three times in wash buffer (Tris-buffered saline containing 0.05% Tween-20 detergent). The bound proteins were finally eluted using elution buffer (21028, Thermo Scientific) and analyzed by IB. To detect the ubiquitination of a specific protein after IP, the beads were washed with wash buffer containing 0.1% SDS and eluted by boiling in 1× SDS loading buffer (P0015, Beyotime). Purified TRIB2 (ab186094), βTubulin (ab164310), and GSK3 (ab60863) were purchased from Abcam, and a purified PCBP2 (H00005094-P01) protein was purchased from Abnova (Taiwan). The antibodies used in co-IP were anti-TRIB2 (ab117981, 1:50 (IP) or 1:1000 (IB), Abcam), anti-PCBP2 (ab184962, 1:50 (IP) or 1:1000 (IB), Abcam), anti-GPX4 (ab125066, 1:100 (IP) or 1:1000 (IB), Abcam), anti-FLAG (14793 or 8146, 1:50 (IP) or 1:1000 (IB), CST), anti-HA (3724 or 2367, 1:50 (IP) or 1:1000 (IB), CST), anti-βTubulin (ab179511, 1:1000 (IB), Abcam), anti-GSK3 (ab32391, 1:5000 (IB), Abcam), and IgG (A7028 or A7016, 1:50 (IP), Beyotime).

### Lysosome and proteasome isolation

Lysosomes were isolated by a lysosome isolation kit (ab234047, Abcam) according to the manufacturer’s instructions. The presence of the lysosomes and the purity of the collected fractions were verified using IB with antibodies against LAMP2 (a lysosome marker) as a positive control and Calnexin (an endoplasmic reticulum marker) as a negative control. Proteasomes were isolated by a proteasome isolation kit (539176, Sigma) according to the manufacturer’s instructions. The presence of the proteasome was verified using IB with specific antibodies against PSMB3 (ab230024, 1:1000, Abcam) and PSMB5 (DF6728, 1:1000, Affinity). Affinity beads and control beads were used to isolate the proteasome and be a negative control, respectively.

### Proximity ligation assay (PLA)

The accurate detection of protein interactions was realized with Duolink in situ PLA probe kits (DUO92004, DUO92002, and DUO92008, Sigma). Briefly, cells were seeded on coverslips and allowed to attach overnight. After fixation in 4% paraformaldehyde, the cells were blocked and incubated overnight with primary antibodies: anti-TRIB2 (ab117981, 1:50, Abcam), anti-PCBP2 (184962, 1:250, Abcam), anti-HA (3724 or 2367, 1:100, CST), anti-FLAG (14793 or 8146, 1:100, CST), anti-βTrCP (11984, 1:100, CST), and anti-PSMB5 (DF6728, 1:100, Affinity; or ab167341, 1:100, Abcam). The average PLA signal intensity of each cell was calculated by ImageJ software.

### Measurements of PSMB5 activity and cell viability

PSMB5 activity was measured by an Amplit fluorimetric proteasome 20S activity assay kit (AAT-13456, AAT Bioquest, Sunnyvale, CA, USA). The absorbance was measured using a fluorescence spectrometer (with an emission wavelength of 525 nm and excitation wavelength of 490 nm). Relative cell viability of the cells was measured by an in vitro neutral red-based toxicology assay kit (TOX-4, Sigma) according to the manufacturer’s instructions. Cell viability was quantified by subtracting the background absorbance at 690 nm from the 540 nm measurement, with the control cells arbitrarily set to 100%.

### Measurements of HIF1α activity

HIF1α luciferase reporter plasmids were cotransfected with Renilla luciferase reporters into cells for treatment. The cells were lysed 24 h post transfection, and luciferase activity was measured using a dual luciferase reagent from Promega (Madison, WI, USA).

### Mass spectrometry (MS)

To reveal the proteins possibly interacting with TRIB2, the immunoprecipitates isolated by the anti-TRIB2 antibodies (ab117981, 1:50, Abcam) were analyzed using a capillary electrophoresis/nanoliquid chromatography (Nano-LC) system coupled with an electrospray ionization and quadrupole-time-of-flight mass spectrometer (ESI-QTOF-MS, Bruker Daltonics, Leipzig, Germany). An internal MASCOT2.4.1 server (Matrix Science, Boston, MA, USA; http://www.matrixscience.com/) and the Swiss-Prot database were employed to identify peptides. The MS data were deposited in ProteomeXchange under accession No. PXD020325 (Username: reviewer65533@ebi.ac.uk, password: 3VfYRglI). The possible existence of PCBP2 in TRIB2 IPs was verified by electrophoresis followed by staining with Coomassie blue dye.

### Statistical analysis

Data were presented as mean ± SD. All the data were normally distributed. Sample size was chosen according to previous reports^6,8^. Tests to examine the differences between groups included Student’s *t*-test and one-way ANOVA. The correlation of TRIB2 and PCBP2 expression in TMA was analyzed by Spearman’s rank correlation. A *P* < 0.05 was regarded as statistically significant. Variance was similar between the groups that were being statistically compared.

## Results

### TRIB2 regulates Ub levels via the proteasome

We first evaluated the specificity of the anti-TRIB2 antibodies. Exogenous FLAG-tagged TRIB1, TRIB2, and TRIB3 were ectopically expressed in HEK293T cells. Immunoprecipitation of exogenous TRIB1, TRIB2 and TRIB3 with anti-FLAG antibodies followed by IB using anti-TRIB2 antibodies demonstrated that the antibodies reacted with TRIB2 but not with TRIB1 and TRIB3 (Supplementary Fig. 1a). Because the molecular weights of the 3 TRIB proteins are very close, we scanned the gel from 10 to 180 kDa and found that only a single clean band could be visualized with the anti-TRIB2 antibodies (Supplementary Fig. 1a), indicating that the anti-TRIB2 antibodies used in the study specifically recognize TRIB2.

**Fig. 1:**
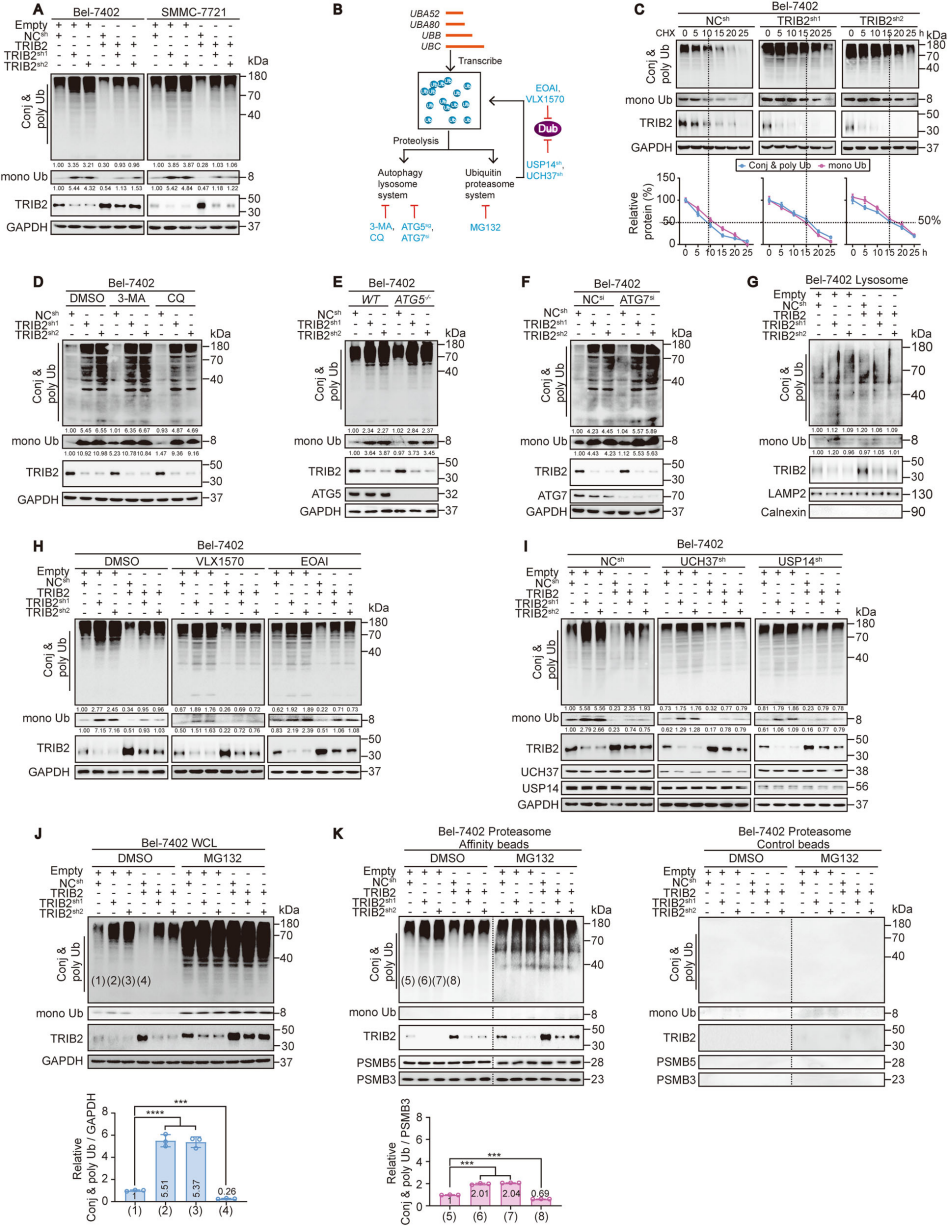
TRIB2 regulates Ub levels via the proteasome. **a** Ub in the control, Bel-7402 and SMMC-7721 cells with TRIB2 knocked down or overexpressed. **b** Schematic representation of Ub homeostasis. **c** CHX chase experiments of Ub in the control and Bel-7402 cells with TRIB2 knocked down. The relative protein levels of conj & poly and mono Ub were normalized to those of GAPDH, respectively, and the “0 h” point was arbitrarily set to 100%. **d**–**f** ALP did not participate in the regulation of Ub levels by TRIB2. Ub in the Bel-7402 cells with or without TRIB2 knocked down after treatment with the ALP inhibitors 3-MA (5 mM for 24 h) and CQ (20 µM, 24 h) (**d**), ATG5 knockout (**e**), or ATG7 knockdown (**f**). **g** Ub in the lysosomes isolated from the same cells as shown in **a**, as measured by IB. The relative protein levels of conj & poly Ub and mono Ub were normalized to those of LAMP2 as calculated by ImageJ software and indicated just below the blots. **h**, **i** TRIB2 did not regulate Ub levels through DUBs. Control and Bel-7402 cells with or without TRIB2 knocked down or overexpressed were treated with DMSO, VLX1570 (20 µM, 24 h), and EOAI (600 nM, 24 h), respectively (**h**), or in the presence or absence of UCH37 and USP14 knocked down (**i**). **j**, **k** TRIB2 regulated Ub levels via the proteasome. Ub levels at WCL (**j**) and in proteasome (**k**) were measured in the same cells shown in **a** and treated with DMSO or MG132 (8 µM, 12 h). The samples in **k** derive from the same experiment and the blots have been processed in parallel using control beads. The levels of conj & poly Ub were normalized to that of GAPDH at WCL (**j**), while normalized to that of PSMB3 in the isolated proteasome (**k**), and the data are graphed below the blots. Data were analyzed by one-way ANOVA and expressed as mean ± SD. ***P < 0.001; ****P < 0.0001. Images of all the immunoblots are representative of three independent experiments. The relative protein levels of the conj & poly Ub and mono Ub were normalized to those of GAPDH as calculated by ImageJ software and indicated just below the blots (**a**, **d**–**f**, **h**, **i**).

To test the relationship between TRIB2 and Ub, we next examined TRIB2 and Ub levels in established cell lines by IB. TRIB2 levels were generally elevated in the liver cancer cell lines (Bel-7402, Bel-7404, HepG2, SMMC-7721 and Huh7 cells) compared to HL-7702 hepatocyte line (Supplementary Fig. 1b). Notably, the levels of conjugated (conj) and poly Ub (represented by bands diffused throughout the lane) and mono Ub (an ~8 kDa band that was distinct from the conj & poly Ub bands) were both negatively associated with the level of TRIB2 (Supplementary Fig. 1b), suggesting that TRIB2 reduces Ub levels. In addition, in Bel-7402 and SMMC-7721 cells, TRIB2 knockdown increased the Ub level, while forced overexpression of TRIB2 decreased the Ub level. The specific effects of TRIB2 knockdown were further proven by the prevention of simultaneous TRIB2 overexpression (Fig. 1a), indicating that TRIB2 might negatively regulate Ub levels in liver cancer cells.

Then, we investigated how TRIB2 regulates Ub. TRIB2 had no effect on the mRNA levels of *UBA52, UBA80, UBB*, and *UBC* in Bel-7402 and SMMC-7721 cells (Supplementary Fig. 1c), excluding the possibility that TRIB2 regulates *Ub* transcription. However, CHX chase experiments demonstrated that the half-lives of both conj & poly Ub and mono Ub can be prolonged by TRIB2 knockdown (Fig. 1c and Supplementary Fig. 1d), suggesting that TRIB2 reduces Ub stability.

Degradation of intracellular proteins is carried out mainly via either the UPS or autophagy-lysosome pathway (ALP) (Fig. 1b, see ref. ^28^). In the presence of the autophagy inhibitor 3-methyladenine (3-MA) or chloroquine (CQ), TRIB2 knockdown elevated Ub levels in Bel-7402 and SMMC-7721 cells (Fig. 1b, d and Supplementary Fig. 1e). At the genetic level, the depletion of ATG5 and ATG7, two key players of autophagy^29^, did not reverse the effects caused by TRIB2 knockdown (Fig. 1b, e, f and Supplementary Fig. 1f, g). As expected, TRIB2 did not regulate Ub levels in lysosomes (Fig. 1g and Supplementary Fig. 1h). These results exclude the involvement of the ALP in the TRIB2-mediated regulation of Ub levels.

Subsequently, we investigated whether TRIB2 regulates Ub levels via the UPS. In this system, DUBs are crucial for Ub recycling (Fig. 1b, see ref. ^12^). However, neither the DUB inhibitor VLX1570 or EOAI (Fig. 1b, h and Supplementary Fig. 1i) nor the depletion of key DUBs, USP14, and UCH37^30^, abolished the effect of TRIB2 in Bel-7402 and SMMC-7721 cells (Fig. 1b, i and Supplementary Fig. 1j), excluding the possibility that TRIB2 regulates Ub via these DUBs. Notably, inhibiting the proteasome with MG132 abrogated the effects of TRIB2 at the whole-cell level (WCL, Fig. 1b, j and Supplementary Fig. 1k). Although conj & poly Ub were present in the isolated proteasome and their levels were negatively associated with the level of TRIB2, mono Ub was undetectable, which is consistent with the fact that mono Ub is an inefficient target for the proteasome because of its compact and globular structure (Fig. 1k and Supplementary Fig. 1l, see ref. ^31^). These results also suggested that the decrease in the mono Ub level at the WCL might have been the result of the decrease in conj & poly Ub levels in the proteasome. By calculating the ratio between conj & poly Ub levels and GAPDH levels at the WCL and between conj & poly Ub levels and PSMB3 levels in the proteasome, we found that the degree to which conj & poly Ub levels were elevated in the proteasome by TRIB2 knockdown was less than that to which they were elevated at the WCL (Fig. 1j, k and Supplementary Fig. 1k, l), suggesting that TRIB2 knockdown dissociates conj & poly Ub from the proteasome. Furthermore, TRIB2 overexpression led to less suppression of conj & poly Ub in the proteasome than at the WCL (Fig. 1j, k and Supplementary Fig. 1k, l), further demonstrating that TRIB2 overexpression facilitates the association of conj & poly Ub with the proteasome. Taken together, these findings suggest that TRIB2 reduces Ub stability possibly by increasing the association between conj & poly Ub and the proteasome.

### PSMB5 within the proteasome is a target of TRIB2

**Fig. 2:**
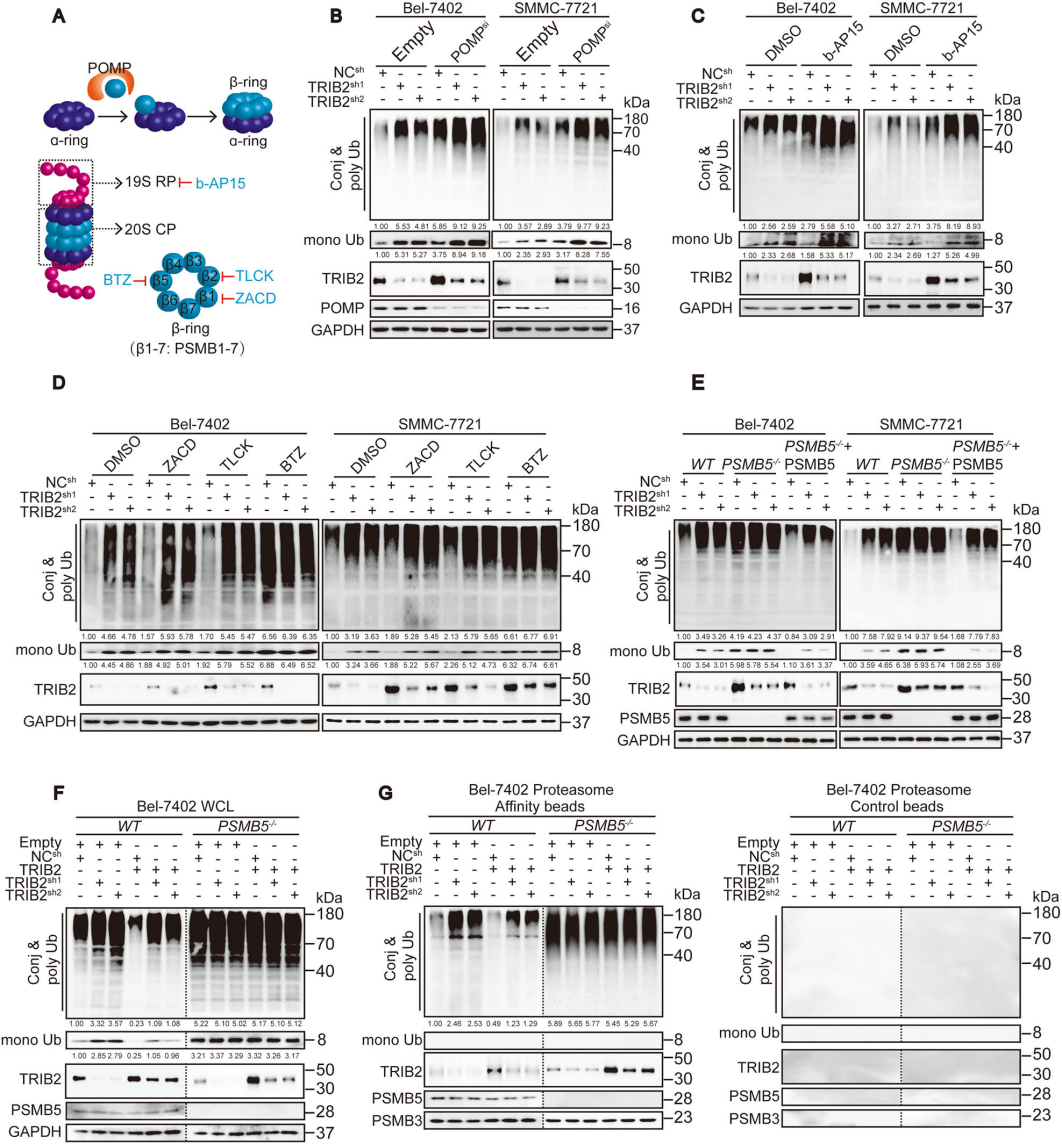
PSMB5 is a target of TRIB2 in the proteasome. **a** Schematic representation of the assembly and composition of the proteasome. **b** TRIB2 affected Ub levels independent of proteasome assembly. Ub levels were measured in the control, Bel-7402, and SMMC-7721 cells with or without TRIB2 knocked down and with or without POMP knocked down. **c**, **d** PSMB5 of 20S CP, but not 19S RP, was involved in the TRIB2-mediated effects on Ub level. Ub levels in the Bel-7402 and SMMC-7721 cells with or without TRIB2 knocked down and treated with DMSO, b-AP15 (5 µM, 24 h) (**c**), ZACD (100 µM, 24 h), TLCK (50 μM, 24 h), or BTZ (100 nM, 24 h) (**d**), as indicated. **e** PSMB5 was critical for TRIB2. Ub was measured in the Bel-7402 and SMMC-7721 cells with or without PSMB5 knocked out or compensatory PSMB5 expression, in the presence or absence of TRIB2 knockdown. **f**, **g** Ub at WCL and in proteasomes isolated from the Bel-7402 cells with TRIB2 knocked down or overexpressed, in the presence or absence of PSMB5. The samples derived from the same experiment using control beads had been processed in parallel. The levels of conj & poly Ub were normalized to that of GAPDH at WCL (**f**), while normalized to that of PSMB3 in the isolated proteasome (**g**). Images of all the immunoblots are representative of three independent experiments. The relative protein levels of the conj & poly Ub and mono Ub were normalized to those of GAPDH as calculated by ImageJ software and indicated just below the blots (**b**–**f**).

Here, we investigated whether TRIB2 leads to a decrease in Ub levels by increasing the proteolytic efficiency of the proteasome. The number and activity of the proteasome are two important factors that affect degradation^32^. Proteasome maturation protein (POMP), which can change the number of proteasomes by affecting their assembly^33^, was knocked down first. However, POMP knockdown did not abolish the effect of TRIB2 on Ub levels in Bel-7402 and SMMC-7721 cells (Fig. 2a, b), excluding the possibility that TRIB2 regulates the proteasome number.

The proteasome is a complex consisting of two 19S regulatory particles (19S RP) and a 20S core pore (20S CP), which contains three different catalytically active sites on subunits PSMB1, PSMB2, and PSMB5 (Fig. 2a, see ref. ^34^). All of the components of proteasome are important for its function. Inhibiting 19S RP by b-AP15 (Fig. 2a, see ref. ^35^) did not abrogate the effects of TRIB2 (Fig. 2c). However, Bel-7402 and SMMC-7721 cells were treated with the 20S CP inhibitor ZACD (targets PSMB1), TLCK (targets PSMB2), or BTZ (targets PSMB5) and only BTZ diminished the effects of TRIB2 (Fig. 2a, d). Similar findings were observed when PSMB5 was knocked out, and this effect that was specifically reversed by the compensatory expression of PSMB5 (Fig. 2e). By comparing conj & poly Ub levels at the WCL and in the proteasome, we found that the effects by which TRIB2 stimulated the association of conj & poly Ub with the proteasome were also indispensable for PSMB5 expression (Fig. 2f, g and Supplementary Fig. 2a, b). Unexpectedly, the PLA experiments showed no direct interaction between TRIB2 and PSMB5; however, an obvious interaction between TRIB2 and E3 βTrCP was observed (Supplementary Fig. 2c, see refs. ^8,36^).

### PCBP2 is essential for TRIB2-mediated regulation of PSMB5 activity

**Fig. 3:**
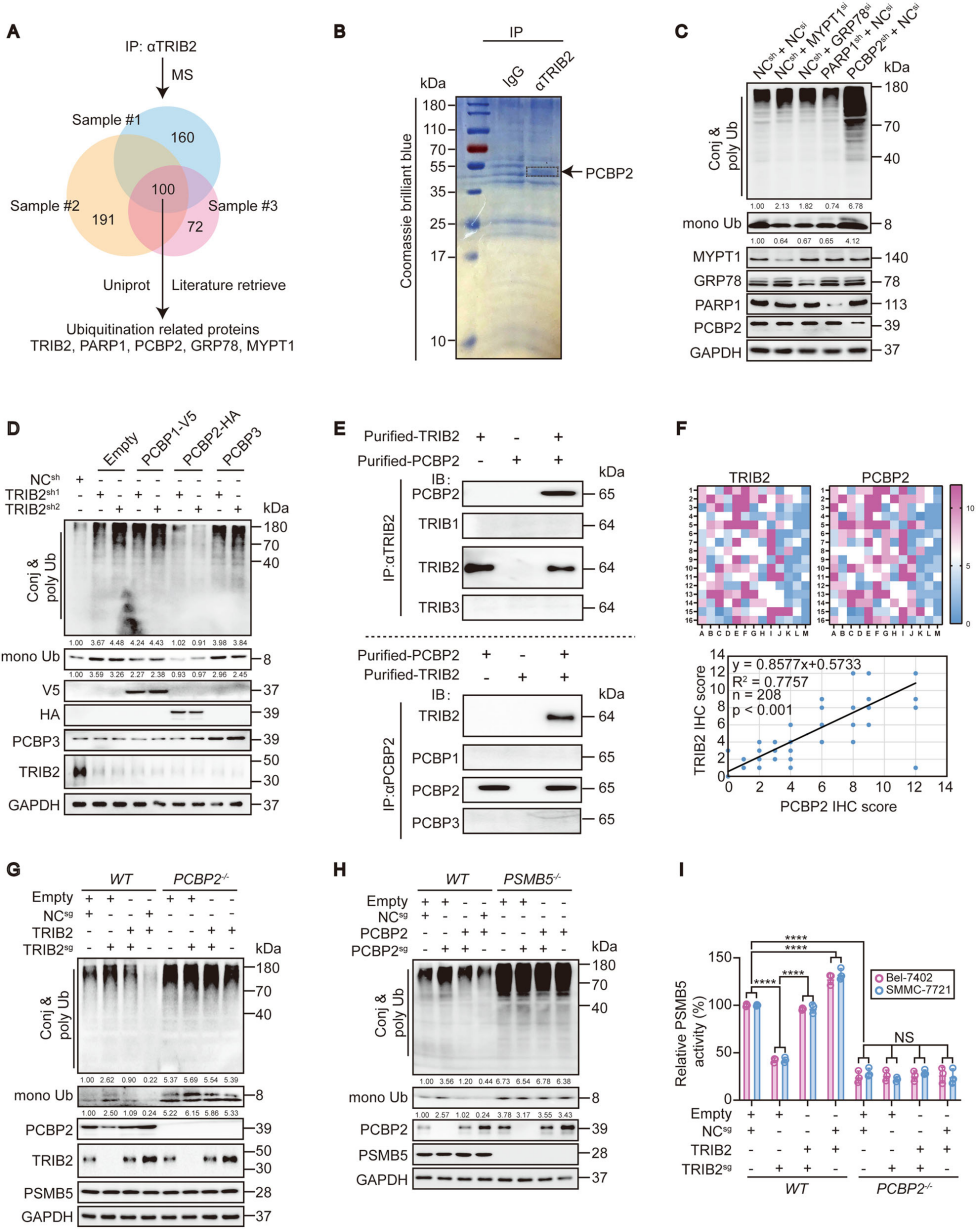
PCBP2 is essential for TRIB2 to regulate PSMB5 activity. **a** Venn diagram showing three biologically independent MS results from the Bel-7402 cell immunoprecipitation with anti-TRIB2 antibodies. The candidates were further screened by UniProt online software and the literature to identify potential ubiquitination-related proteins. **b** Representative Coomassie blue stain image of the Bel-7402 cell immunoprecipitates pulled down by anti-TRIB2 or IgG antibodies (*n* = 3). **c** Ub in the control and Bel-7402 cells with MYPT1, GRP78, PARP1, or PCBP2 knocked down. **d** Ub in the control and Bel-7402 cells overexpressing PCBP1, PCBP2, or PCBP3 with or without TRIB2 knocked down. **e** Reciprocal co-IP results of purified TRIB2 and PCBP2 proteins in vitro. TRIB1, TRIB3, PCBP1, and PCBP3 were parallel examined to exclude non-specificity. **f** Positive correlation between PCBP2 and TRIB2 in the liver cancer specimens. TMA was performed by IHC using anti-PCBP2 and anti-TRIB2 antibodies (*n* = 208). The data were analyzed by Spearman’s rank correlation. **g** The effects of TRIB2 on Ub level were PCBP2-dependent. Ub in the control and Bel-7402 cells with or without TRIB2 knocked out or overexpressed, in the presence or absence of PCBP2 knocking out. **h** PCBP2 regulated Ub via PSMB5. Ub in Bel-7402 cells with PCBP2 and PSMB5 knocked out or overexpressed, as indicated. **i** TRIB2 regulated PSMB5 activity via PCBP2. A proteasome activity assay kit (AAT Bioquest) was used to evaluate PSMB5 activity in the Bel-7402 and SMMC-7721 cells under the same treatment as indicated in **g** (*n* = 3). Data were analyzed by one-way ANOVA and expressed as mean ± SD. ****P < 0.0001; NS non-significance. Images of all the immunoblots are representative of three independent experiments. The relative protein levels of the conj & poly Ub and mono Ub were normalized to those of GAPDH as calculated by ImageJ software and indicated just below the blots (**c**, **d**, **g**, **h**).

To identify the protein that is essential for the targeting of PSMB5 by TRIB2, materials bound to TRIB2 immunoprecipitates from the lysates of Bel-7402 cells were analyzed by MS (Fig. 3a). To identify Ub-associated proteins, 100 common candidates obtained from three independent biological replicates were further narrowed down according to the literature and with UniProt online software (https://www.uniprot.org). Four proteins, i.e., GRP78^37^, MYPT1^38^, PARP1^39^, and PCBP2^21^, remained as candidates. The bound materials that immunoprecipitated with the anti-TRIB2 antibodies were stained with Coomassie blue dye, and a 35–55 kDa band distinct from the band observed following immunoprecipitation with control IgG antibodies was seen, which was speculated to be the PCBP2 protein (39 kDa) (Fig. 3b). Functionally, silencing of three proteins, other than PARP1, in Bel-7402 cells led to increased levels of conj & poly Ub; however, silencing PCBP2 caused a simultaneous increase in mono Ub, which was similar to what was observed in the TRIB2 knockdown experiment (Figs. 1a and 3c). To further clarify the role of PCBP2, we tested the effect of its analogs. In contrast to PCBP2, neither PCBP1 nor PCBP3 reversed the effect of TRIB2 knockdown in Bel-7402 cells (Fig. 3d), confirming that PCPB2 is essential for the TRIB2-mediated regulation of Ub.

The correlation between TRIB2 and PCBP2 was then investigated. First, we evaluated the specificity of the purified TRIB2 and PCBP2 proteins. The anti-TRIB1, anti-TRIB2, and anti-TRIB3 antibodies recognized their corresponding endogenous proteins in Bel-7402 cells (Supplementary Fig. 3a); however, only the anti-TRIB2 antibodies reacted with the purified TRIB2 protein (Fig. 3e). Similarly, the specificity of the purified PCBP2 proteins was verified (Fig. 3e and Supplementary Fig. 3a). We also confirmed that there was not an ancillary factor in the purified TRIB2 and PCBP2 proteins, because no interaction between purified unrelated wheat germ-synthesized PCBP2 and βTubulin proteins and between unrelated Sf9 cell-synthesized TRIB2 and GSK3 proteins was detected (Supplementary Fig. 3b). Then, reciprocal co-IP of purified TRIB2 and PCBP2 proteins (Fig. 3e) and of exogenous TRIB2-FLAG and PCBP2-HA from HEK-293T cells (Supplementary Fig. 3c) was performed. These experiments clarified the interaction between TRIB2 and PCBP2. The PLA experiments further demonstrated the direct interaction between these proteins (Supplementary Fig. 3d). A TMA was then performed, and Spearman’s rank correlation coefficient analysis showed a significant positive correlation between the IRSs of TRIB2 and PCBP2 (Fig. 3f and Supplementary Fig. 3e). This close relationship was verified by the GEPIA online database (http://gepia.cancer-pku.cn/) (Supplementary Fig. 3f). Importantly, TRIB2 lost its ability to suppress Ub when PCBP2 was knocked out (Fig. 3g). However, the opposite outcome was not found when the situation was reversed (Supplementary Fig. 3g), indicating that PCBP2 acts solely downstream of TRIB2.

Next, we examined whether the function of PCBP2 relies on PSMB5. As shown in Fig. 3h, PCBP2 failed to reduce Ub levels when PSMB5 was knocked out in Bel-7402 cells. Combined with the results of the PLA experiments in both Bel-7402 and SMMC-7721 cells (Supplementary Fig. 3h), this finding suggests that PCBP2 regulates the Ub level via direct interaction with PSMB5. Notably, TRIB2 and PCBP2 regulated the activity but not the expression of PSMB5 (Fig. 3g–i and Supplementary Fig. 3g, i).

### TRIB2 suppresses the K48-ubiquitination of PCBP2 and reduces global K48-Ub levels

**Fig. 4:**
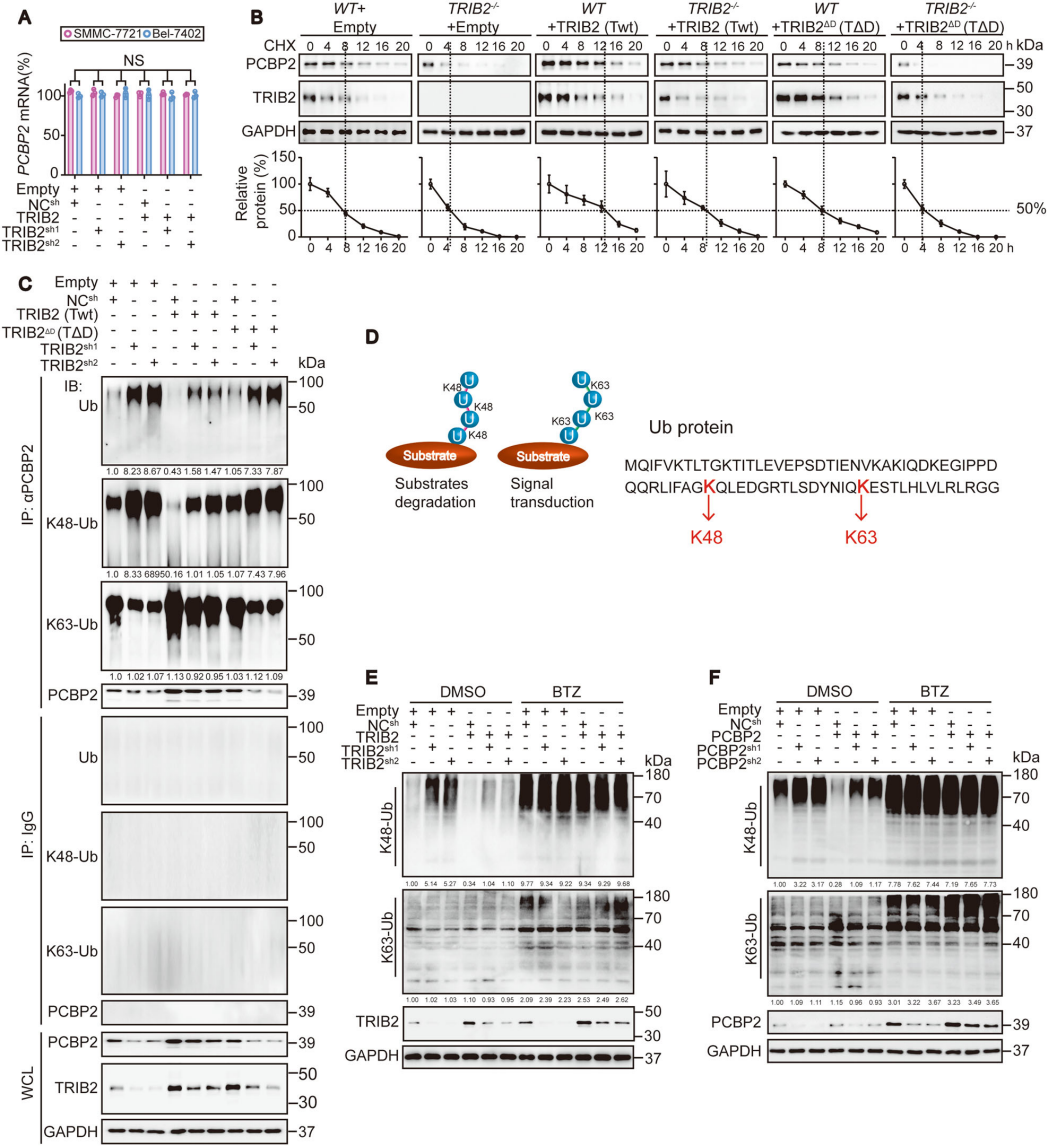
TRIB2 boosts PCBP2 and reduces the global K48-Ub level. **a** mRNA levels of *PCBP2* in the control, Bel-7402, and SMMC-7721 cells with TRIB2 knocked down or overexpressed, as measured by qRT-PCR (*n* = 3). Data were analyzed by one-way ANOVA and expressed as mean ± SD. NS non-significance. **b** CHX chase experiments of PCBP2 in the control and Bel-7402 cells with or without TRIB2 knocked out in the presence or absence of TRIB2 (Twt) or TRIB2^ΔD^ (TΔD). The relative protein levels of PCBP2 were normalized to those of GAPDH, and the “0 h” point was arbitrarily set to 100%. Data were expressed as mean ± SD. **c** K48-ubiquitination, K63-ubiquitination and total-ubiquitination of PCBP2, as immunoprecipitated by anti-PCBP2 antibodies for the indicated treatment in Bel-7402 cells, and measured by anti-K48, anti-K63, and anti-total Ub antibodies. The relative K48-Ub, k63-Ub, and total-Ub of PCBP2 were normalized to those of PCBP2 in the PCBP2-IPs and indicated below the blots. **d** Schematic representation of the Ub K48 and K63 linkages. **e**, **f** Global K48-Ub level was specifically targeted by TRIB2 and PCBP2. K48-Ub and K63-Ub levels were evaluated by anti-K48 and anti-K63 Ub antibodies in the Bel-7402 cells with TRIB2 (**e**) or PCBP2 (**f**) knocked down or overexpressed and treated cells with DMSO or BTZ (100 nM, 24 h). The relative protein levels of the global K48-Ub and K63-Ub were normalized to those of GAPDH as calculated by ImageJ software and indicated just below the blots (**e**, **f**). Images of all the immunoblots are representative of three independent experiments.

Although PCBP2 acts downstream of TRIB2 (Fig. 3g and Supplementary Fig. 3g), the regulatory mechanism remains unknown. On the basis of the findings of the qPCR and CHX chase experiments, we suggest that TRIB2 might regulate the protein stability of PCBP2 but not *PCBP2* mRNA (Fig. 4a, b). Considering the close relationship between TRIB2 and the UPS, we explored the ubiquitination level of PCBP2 and found that TRIB2 significantly decreased PCBP2 ubiquitination (Fig. 4c and Supplementary Fig. 4). The effects of ubiquitination vary on the basis of Ub linkages^40^, among which the Lys48 (K48) and Lys63 (K63) linkages are the best characterized forms related to substrate degradation and signal transduction, respectively (Fig. 4d, see ref. ^41^). We found that TRIB2 suppressed the total-ubiquitination and K48-ubiquitination but not the K63-ubiquitination of PCBP2. In contrast, TRIB2 positively regulated PCBP2 expression (Fig. 4c and Supplementary Fig. 4). These results suggested that TRIB2 upregulates PCBP2 largely by reducing its K48-ubiquitination.

Inspired by the finding that TRIB2 decreases PCBP2 K48-ubiquitination, we speculated that TRIB2 decreases the global K48-Ub level. To test this supposition, we evaluated the levels of K48-Ub and K63-Ub with specific antibodies. Indeed, TRIB2 reduced the global K48-Ub level, and this effect was effectively blocked by the PSMB5 inhibitor BTZ. Unfortunately, TRIB2 had no effects on the global K63-Ub level (Fig. 4e). As a downstream of TRIB2, PCBP2 exhibited a similar function, reducing the global K48-Ub level, which was also PSMB5-dependent (Fig. 4f). Therefore, a model in which TRIB2 cooperates with and stimulates PCBP2 to reduce the global K48-Ub level was established.

### The DQLVPD element and the KH3 domain are essential for the TRIB2–PCBP2 interaction and its function

**Fig. 5:**
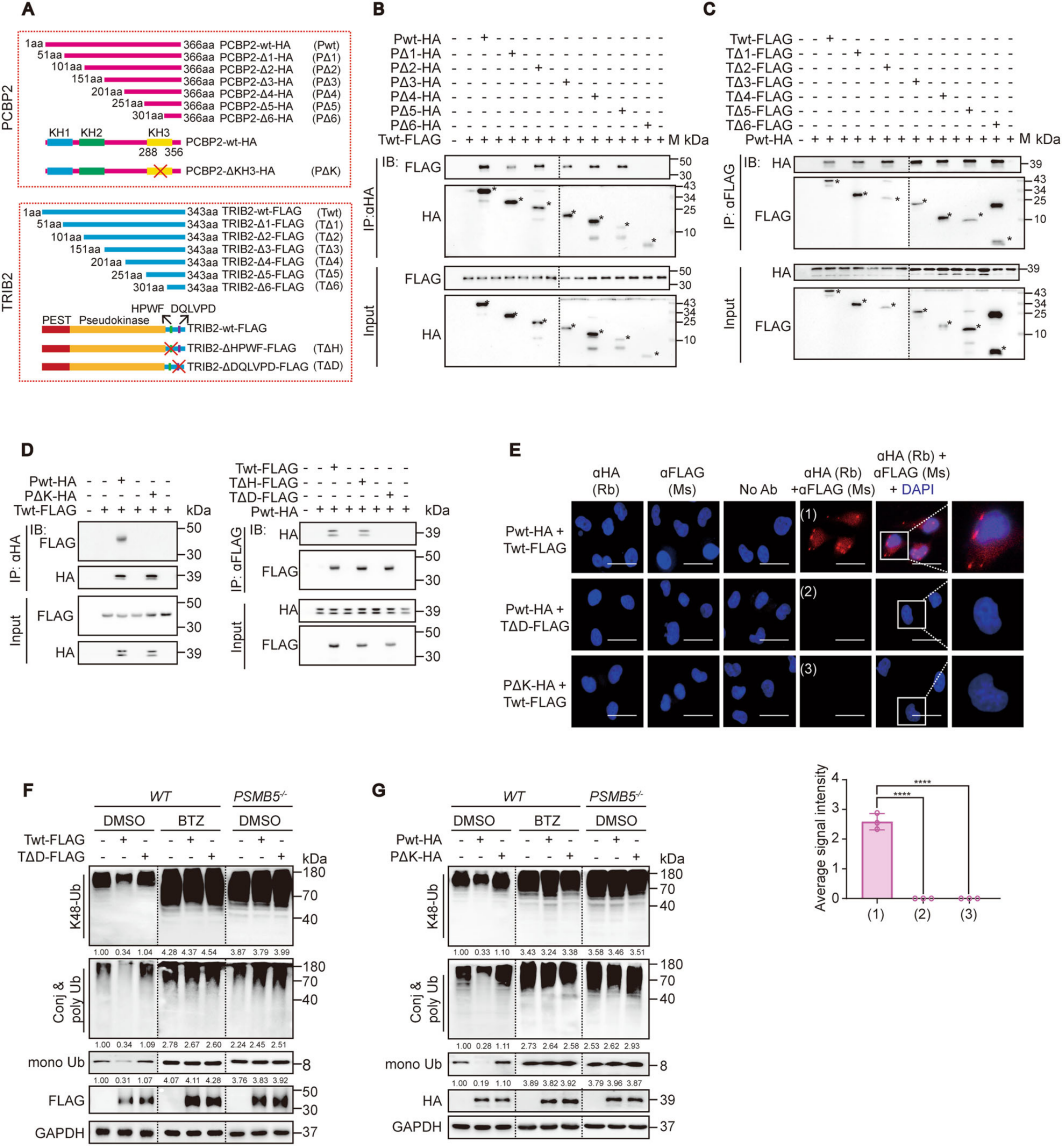
The DQLVPD element and the KH3 domain are essential for TRIB2–PCBP2 interaction and function. **a** Schematic representation of the construction of the PCBP2-expressing and TRIB2-expressing plasmids. **b**, **c** co-IP were performed between TRIB2 and PCBP2 and their mutants in HEK-293T cells as indicated. The asterisks indicate the specific bands that represent truncated versions of TRIB2 and PCBP2. M, indicates protein ladder. **d** The DQLVPD element and the KH3 domain were essential for TRIB2–PCBP2 binding. co-IP were performed between indicated TRIB2 and PCBP2 constructs in HEK-293T cells. **e** Direct TRIB2-PCBP2 interaction relies on the DQLVPD element and KH3 domain. Interactions were measured by PLA experiments with Bel-7402 cells expressing the TRIB2 and PCBP2 as indicated (*n* = 3); Scale bar = 25 µm. The average PLA signals per cell were graphed below. Data were analyzed by Student’s *t*-test and expressed as mean ± SD. ****P < 0.0001. **f**, **g** The TRIB2 DQLVPD element and PCBP2 KH3 domain were essential to reduce K48-Ub levels. K48-Ub and total-Ub levels were measured by anti-K48-Ub and anti-Ub antibodies in the control and Bel-7402 cells overexpressing TRIB2 (Twt or TΔD) (**f**) or PCBP2 (Pwt or PΔK) (**g**) in the treatment of DMSO, BTZ (100 nM, 24 h) or PSMB5 knockout. The relative protein levels of global K48-Ub, conj & poly Ub and mono Ub were normalized to those of GAPDH as calculated by ImageJ software and indicated just below the blots (**f**, **g**). Images of all the immunoblots are representative of three independent experiments.

To further investigate the structural basis of the TRIB2–PCBP2 interaction, we constructed a series of plasmids expressing truncated versions of PCBP2 (PΔ1–6) and TRIB2 (TΔ1–6) (Fig. 5a). In contrast to PΔ6, WT-PCBP2 (Pwt) and PΔ1–5 bound WT-TRIB2 (Twt) in HEK-293T cells (Fig. 5b and Supplementary Fig. 5a), suggesting that the amino acid (a.a.) sequences in the PΔ5 and PΔ6 mutants are essential for the TRIB2–PCBP2 interaction. Compared to PΔ5, PΔ6 lacks a 251–301 a.a. region, within which an overlapping KH3 domain was reported (Fig. 5a, see ref. ^42^). All TRIB2 mutants interacted with Pwt (Fig. 5c and Supplementary Fig. 5b), suggesting that TΔ6 contains the essential interaction site. As shown in Fig. 5a, TΔ6 includes two special domains, a DQLVPD element known to interact with E3 ligase and an HPWF motif that binds with MEK1^43,44^. We then constructed PCBP2 and TRIB2 variants lacking either the KH3 domain, DQLVPD element, or HPWF motif (Fig. 5a). Reciprocal co-IP demonstrated that the DQLVPD element of TRIB2 and the KH3 domain of PCBP2 are essential for the TRIB2–PCBP2 interaction (Fig. 5d), and PLA experiments corroborated this result (Fig. 5e).

Next, we examined whether the DQLVPD element and the KH3 domain are functionally important. As expected, like the deletion of the KH3 domain in PCBP2 (PΔK) (Fig. 5g), the deletion of the DQLVPD element in TRIB2 (TΔD) failed to decrease the PSMB5-dependent global K48-, conj & poly and mono Ub levels in the Bel-7402 cells (Fig. 5f). Regarding the regulation of PCBP2, Twt but not TΔD reversed the decrease in the half-life of PCBP2 in TRIB2^−/−^ Bel-7402 cells (Fig. 4b). Moreover, the DQLVPD element was also essential for the decrease in the K48-ubiquitination of PCBP2 by TRIB2 (Fig. 4c). In general, the DQLVPD–KH3 interaction is the structural basis for the cooperation between TRIB2 and PCBP2.

### PCBP2 and TRIB2 maintain cell viability via GPX4 under OS

Depletion of PCBP2 reduces the prolyl hydroxylation of HIF1α (HIF1α-OH); it also induces total HIF1α expression after treatment with MG132 and the iron chelator DFO^45^. To further verify that PCBP2 acts downstream of TRIB2, we ectopically expressed PCBP2 driven by the CMV promoter in TRIB2^−/−^ Bel-7402 and SMMC-7721 cells. We speculated that because PCBP2 was destabilized when TRIB2 was depleted (Fig. 4b, c), the level of intracellular PCBP2 that would be maintained by the overexpression of PCBP2 in TRIB2^−/−^ cells would be lower than that in WT cells; however, the level of PCBP2 was very close to the level observed before TRIB2 knockout (Fig. 7a and Supplementary Figs. 6a, 7a). Under such conditions, nuclear extracts from TRIB2^−/−^ Bel-7402 cells were probed for HIF1α-OH and total HIF1α. HIF1α-OH and total HIF1α levels were reduced and increased, respectively, in TRIB2^−/−^ Bel-7402 cells, and these effects were reversed by overexpressing PCBP2 (Supplementary Fig. 6b). Together with the HIF1α luciferase reporter data (Supplementary Fig. 6c), these findings further suggest that PCBP2 is a downstream effector of TRIB2.

**Fig. 6:**
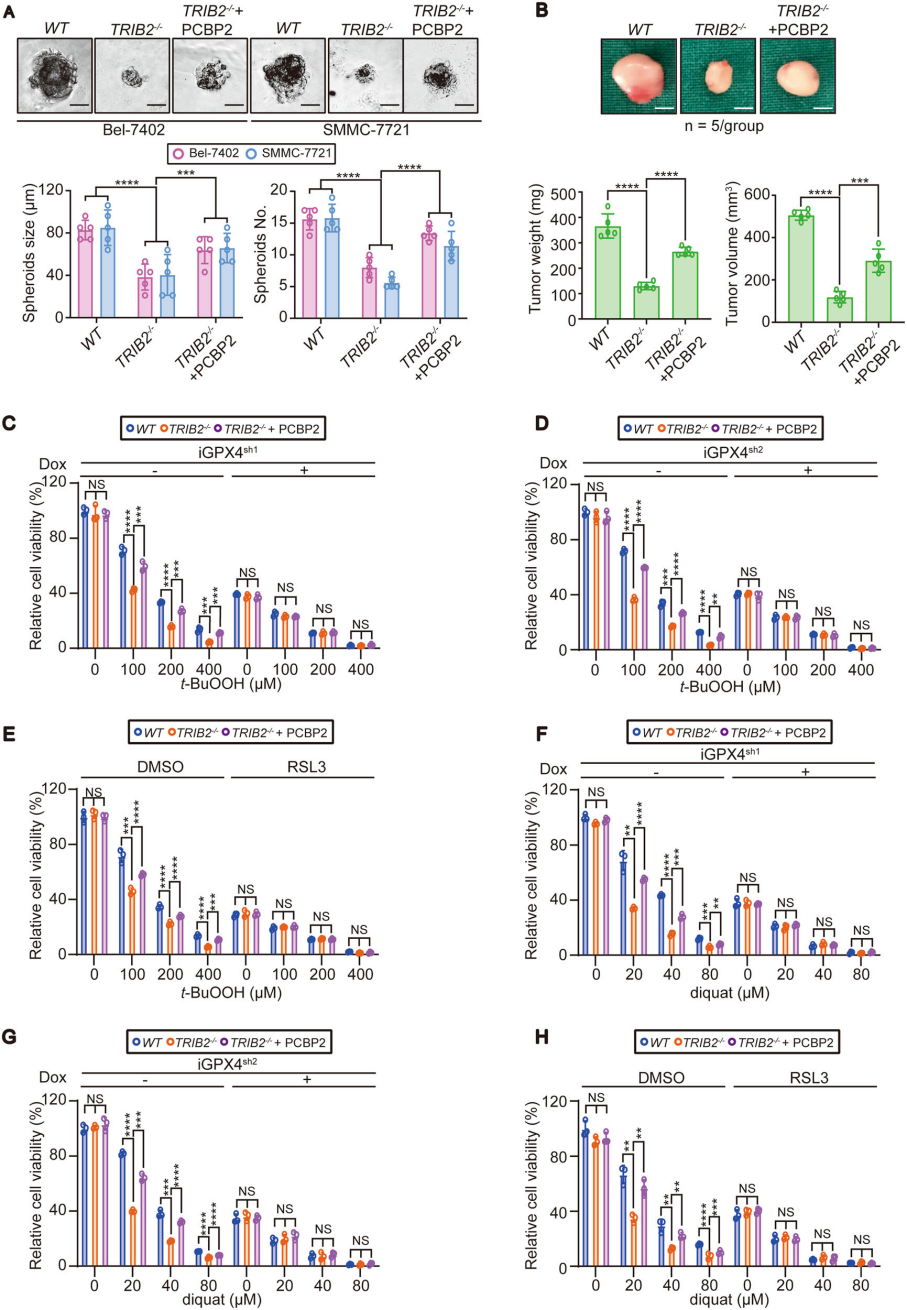
PCBP2 and TRIB2 maintain cell viability via GPX4 under OS. **a** Spheroid formation by the Bel-7402 and SMMC-7721 cells with or without TRIB2 knocked out, in the presence or absence of overexpressed PCBP2 (*n* = 3); Scale bar = 30 µm. Data were analyzed by one-way ANOVA and expressed as mean ± SD. ***P < 0.001; ****P < 0.0001. **b** Tumor growth in athymic mice inoculated with Bel-7402 cells with or without TRIB2 knocked out in the presence or absence of overexpressed PCBP2 (*n* = 5/group); Scale bar = 4 mm. The data were analyzed by Student’s *t*-test and expressed as mean ± SD. ***P < 0.001; ****P < 0.0001. **c**–**h** Inhibition of GPX4 diminished the ability of TRIB2 and PCBP2 to protect cells against OS. Bel-7402 cells transfected with Dox-inducible shRNA targeting GPX4 (iGPX4^sh1^ (**c**, **f**); iGPX4^sh2^ (**d**, **g**)) were pretreated with or without Dox (700 ng/ml, 24 h) (**c**, **d**, **f**, **g**). Bel-7402 cells were also pretreated with or without RSL3 (5 µM) for 5 h (**e**, **h**). Then, the cells were exposed to the indicated concentration of *t*-BuOOH (**c**–**e**) or diquat (**f**–**h**) for another 24 h. Cell viability was determined and calculated as described in the “Materials and methods” section (*n* = 3). The data were analyzed by one-way ANOVA and expressed as mean ± SD. **P < 0.01; ***P < 0.001; ****P < 0.0001. NS non-significance.

TRIB2 and PCBP2 play crucial roles in various dieases^22,23,46,47^, but their cooperative effects in liver cancer has not been fully explained. In vitro and in vivo cell function experiments showed that knocking out TRIB2 reduced the size and number of spheroids generated by Bel-7402 and SMMC-7721 cells in 3D culture (Fig. 6a and Supplementary Fig. 6a) and attenuated the weight and volume of tumors generated by Bel-7402 cells in athymic mice (Fig. 6b). In addition, all of these effects were reversed by treatment with PCBP2, suggesting that PCBP2 and TRIB2 increase liver cancer cell viability.

Both TRIB2 and PCBP2 have crucial roles in protecting cells against oxidative damage^19,20^. However, whether these functions play important roles in maintaining cell viability is unknown. To answer this question, *t-*BuOOH, an oxidizing agent that generates lipid peroxide^48^, was used to induce OS in cells. TRIB2 deficiency in Bel-7402 and SMMC-7721 cells led to dose-dependent decreases in cell survival after exposure to various concentrations of *t*-BuOOH. As expected, PCBP2 largely reversed this effect (Fig. 6c–e and Supplementary Fig. 6e–g). GPX4 is the primary enzyme that defends against OS induced by *t*-BuOOH^49^. Blocking GPX4 via either gene inhibition mediated by two distinct Dox-inducible shRNAs (Fig. 6c, d and Supplementary Fig. 6e, f) or treatment with RSL3 (Fig. 6e and Supplementary Fig. 6g), a promising chemical GPX4 inhibitor, diminished the effects of TRIB2 and PCBP2. Similar effects were observed when Bel-7402 and SMMC-7721 cells were treated with diquat, another oxidizing agent that generates superoxide (Fig. 6f–h and Supplementary Fig. 6h–j, see ref. ^50^). Thus, TRIB2 and PCBP2 maintain liver cancer cells viability via GPX4 to protect them against OS. Of note, TRIB2 and PCBP2 were also important for maintaining the viability of lung cancer A549 cells via GPX4 (Supplementary Fig. 6k, l), indicating that these effects might not be restricted to liver cancer cells.

### TRIB2 and PCBP2 reduce the K48-ubiquitination of GPX4 to maintain its expression

**Fig. 7:**
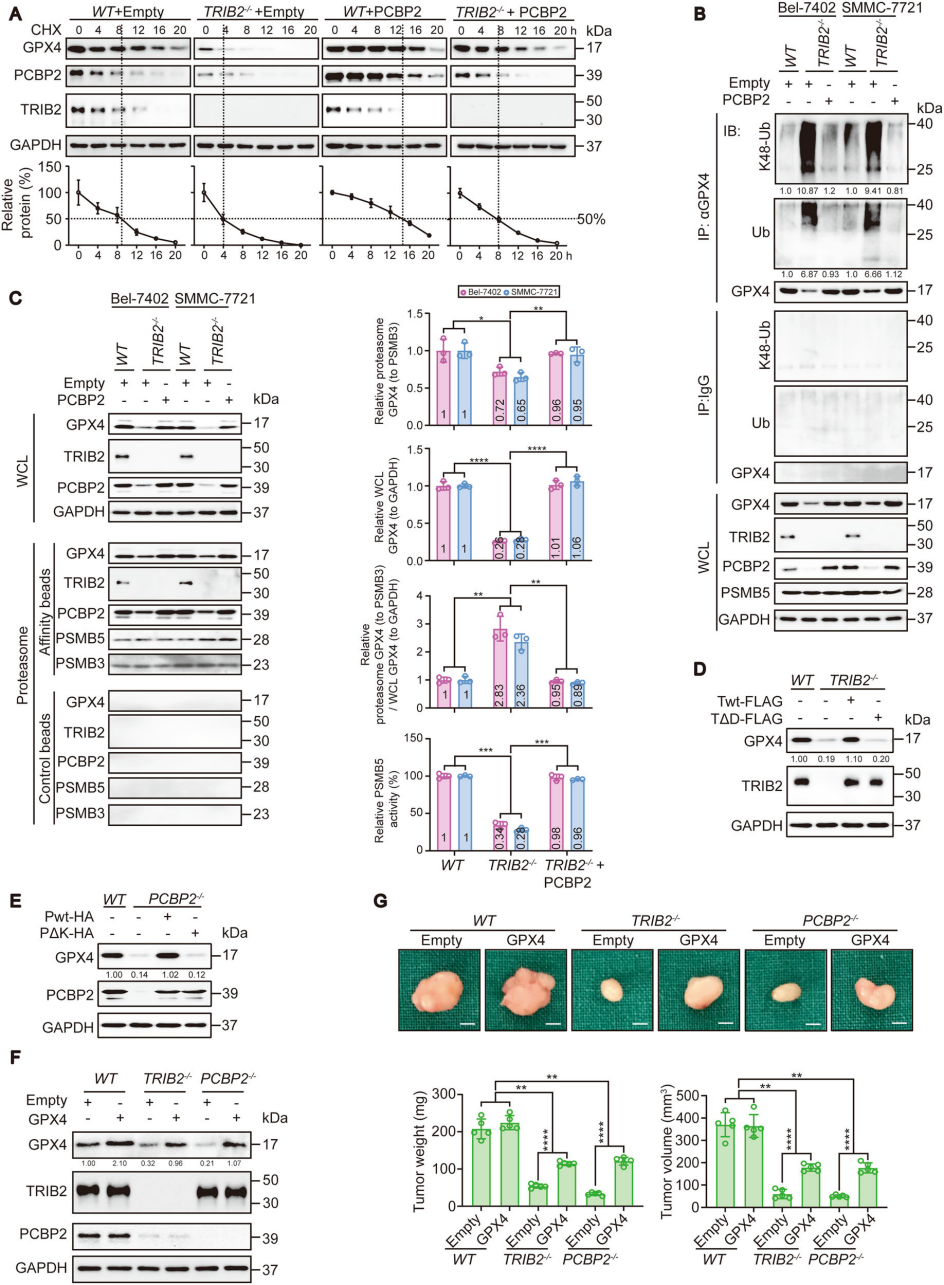
TRIB2 and PCBP2 reduce K48-ubiquitination of GPX4. **a** CHX chase experiments of GPX4 in the control and Bel-7402 cells with TRIB2 knocked out, with or without the simultaneous overexpression of PCBP2. The relative protein levels of GPX4 were normalized to those of GAPDH, and the “0 h” point was arbitrarily set to 100%. **b** TRIB2 and PCBP2 regulated GPX4 ubiquitination. GPX4 or its K48-ubiquitination and total-ubiquitination level in the WCL of Bel-7402 and SMMC-7721 cells with or without TRIB2 knockout in the presence or absence of ectopic expression of PCBP2. The K48-Ub and total-Ub of GPX4 were normalized to that of GPX4 in the GPX4-IPs by ImageJ and indicated below the blots. **c** PCBP2 acts as the downstream of TRIB2 to prevent the association between GPX4 and proteasome in Bel-7402 and SMMC-7721 cells. The relative GPX4 levels were normalized to PSMB3 in proteasome and normalized to GAPDH at WCL. The relative GPX4 levels were also calculated as the ratio between the one in proteasome and the one at WCL. Besides, the PSMB5 activity was also parallel examined at WCL. Data were analyzed by one-way ANOVA and expressed as mean ± SD. *P < 0.05; **P < 0.01; ***P < 0.001; ****P < 0.0001. **d**, **e** The TRIB2 DQLVPD element and PCBP2 KH3 domain are critical to maintain GPX4 expression. GPX4 was measured by IB in the Bel-7402 cells transfected with the indicated plasmids. The relative GPX4 levels were normalized to that of GAPDH and indicated below the blots. **f** GPX4 in control and Bel-7402 cells with or without TRIB2 or PCBP2 knocked out in the presence or absence of overexpressed GPX4. The relative GPX4 levels were normalized to that of GAPDH and indicated below the blots. **g** Tumor growth in athymic mice inoculated with the same cells as shown in **f**. The weight and volume of a tumor are presented below the representative tumor image (*n* = 5/group); Scale bar = 3 mm. Data were analyzed by one-way ANOVA and expressed as mean ± SD. **P < 0.01; ****P < 0.0001. Images of all the immunoblots are representative of three independent experiments.

Next, we investigated whether and how TRIB2 and PCBP2 regulate GPX4. qPCR and CHX chase experiments suggested that TRIB2 and PCBP2 regulated GPX4 protein stability rather than *GPX4* mRNA (Fig. 7a and Supplementary Figs. 6m, 7a). We found that overexpressing PCBP2 mitigated the TRIB2-knockout-induced reduction in GPX4 expression. Moreover, the regulatory effect of TRIB2 knockout on total- and K48-ubiquitination of GPX4 was reversed (Fig. 7b). The increase in the association of GPX4 with the proteasome accompanied by the decrease in PSMB5 activity in TRIB2^−/−^ Bel-7402 and SMMC-7721 cells compared to WT cells was also reversed by PCBP2 overexpression (Fig. 7c). The results suggest that the TRIB2–PCBP2 axis regulates GPX4 stability, possibly via the proteasome.

In the absence of TRIB2, the level of Ub was increased because PSMB5 was less active (Figs. 1–3). However, the absence of TRIB2 led to destabilization of GPX4, possibly because there was more Ub to make K48-Ub chains (Fig. 7a, b). Is the stability of GPX4 controlled by components in the proteasome other than PSMB5 when Ub is abundantly available? In addition to PSMB5, PSMB1, and PSMB2 are distinct catalytically active sites in the proteasome^34^. Ub, K48-Ub (without other lysine residues except K48), or K63-Ub (without other lysine residues except K63) were overexpressed in Bel-7402 cells. GFP was simultaneously overexpressed to further investigate whether other proteins are also influenced. Similar to TRIB2 deficiency, overexpression of Ub and K48-Ub, but not K63-Ub, reduced GPX4 expression. However, these effects were diminished when PSMB1 and PSMB2 were blocked by the inhibitors TLCK and ZACD. Unfortunately, GFP levels were not as obviously affected as GPX4 levels (Supplementary Fig. 7b, c). These results demonstrate that PSMB1 and PSMB2 are activated to degrade proteins when Ub efflux is increased, such as when PSMB5 is inhibited and that GPX4 might be more sensitive to these effects.

Next, we evaluated whether the DQLVPD element of TRIB2 and the KH3 domain of PCBP2 are critical to regulating GPX4. In TRIB2^−/−^ Bel-7402 cells, the reconstitution of TRIB2 expression by Twt successfully reversed the suppression of GPX4, while TΔD failed to have this effect (Fig. 7d). In addition, compared to Pwt, PΔK was unable to maintain GPX4 expression (Fig. 7e). Functionally, the reduction in the in vivo growth of the tumors formed by the Bel-7402 cells upon the knockout of either TRIB2 or PCBP2 was partially rescued by GPX4 in athymic nude mice (Fig. 7f, g), suggesting that GPX4 is a terminal effector of TRIB2 and PCBP2 to boost liver tumorigenesis.

Although the TRIB2–PCBP2–GPX4 axis is critical for tumorigenesis (Figs. 6 and 7), small-molecule compounds that target this axis have not yet been identified. Afatinib, a small-molecule protein kinase inhibitor that inhibits epidermal growth factor receptor tyrosine kinase, was found to be a TRIB2-destabilizing agent^51^. Treatment of Bel-7402 and SMMC-7721 cells with afatinib significantly decreased TRIB2 and PCBP2 levels (Supplementary Fig. 7d) and markedly suppressed PSMB5 activity (Supplementary Fig. 7e), ultimately leading to elevation of conj & poly Ub, mono Ub and global K48-Ub levels (Supplementary Fig. 7d). Cell viability was also suppressed following afatinib treatment (Supplementary Fig. 7f), indicating that targeting TRIB2 is a potential strategy for treating cancer.

## Discussion

**Fig. 8:**
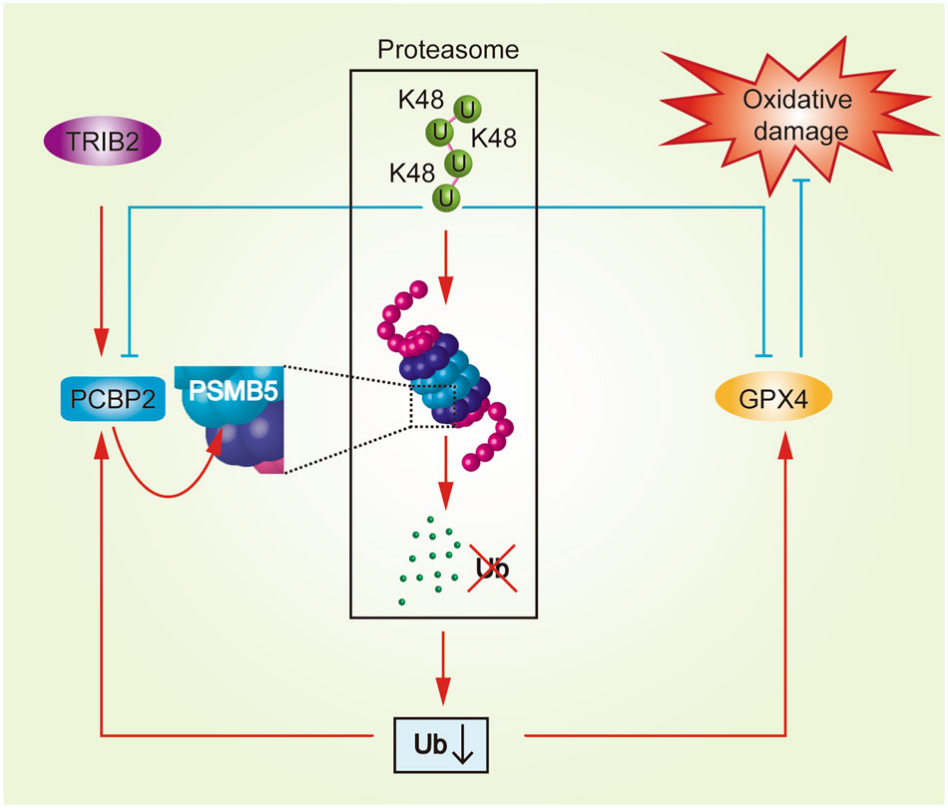
Schematic diagram of the mechanism by which TRIB2 and PCBP2 modulate proteasome function to reduce Ub and protect cells against OS via GPX4. Briefly, TRIB2 cooperates and stimulates PCBP2 to reduce the global K48-Ub level. PSMB5 is the target of these proteins, and its activity is directly promoted by PCBP2. The levels of PCBP2 and GPX4 can be upregulated via suppression of their ubiquitination. Moreover, GPX4 functions as one of the terminal effectors of TRIB2 and PCBP2 to protect liver cancer cells from oxidative damage.

Here, we reveal that in liver cancer cells, PCBP2 facilitates the TRIB2-induced reduction in global K48-Ub levels by elevating the activity of PSMB5, one of the major components of the proteasome that contains active sites. The lack of available Ub eventually prevents the K48-ubiquitination of PCBP2, stabilizing it. On the basis of these findings, a model showing that TRIB2 cooperates with and stimulates PCBP2 to reduce the global K48-Ub level was established. The K48-ubiquitination of GPX4 was also simultaneously blocked, by which prevents OS-induced damage to stimulate liver tumorigenesis (Fig. 8).

Previous studies have implied that TRIB2 acts as a positive modulator of substrate ubiquitination by interacting with E3s^1,26,36^. However, in this study, we revealed that PCBP2, which is not a genuine E3, binds to TRIB2 and regulates K48-dependent Ub degradation via the proteasome. To the best of our knowledge, this is the first study to elucidate the role of TRIB2 in regulating the UPS beyond specifically regulating a single E3. Interestingly, PCBP2 and E3s share a common binding site in TRIB2, i.e., the DQLVPD element. For this reason, we hypothesized that TRIB2 might promote Ub degradation in two ways: via E3s because E3s facilitate Ub conjugation with substrates and engagement with the proteasome and via PCBP2 because it enhances Ub hydrolysis by increasing PSMB5 activity. Although our study revealed an E3-independent pathway by which TRIB2 regulates overall Ub levels, how TRIB2 switches between E3-dependent and PCBP2-dependent pathways to reduce the global K48-Ub level is still unknown and needs to be investigated in our future study.

To date, the vast majority of studies have focused on how K48-ubiquitinated substrates are degraded within the proteasome, and almost no attention has been paid to the degradation of K48-linked Ub itself. Here, we imply that PSMB5 is the main active site that determines the rate of K48-dependent Ub degradation. Phosphorylation of PSMB5 affects proteasome activity directly^52^. It has been demonstrated that protein kinase G (PKG) is capable of affecting phosphorylation and increasing the activity of PSMB5. Notably, PCBP2 is necessary to stimulate cAMP^53^, which can partially activate PKA and PKG^54^. The mechanism by which PCBP2 boosts PSMB5 function might be explained by this effect.

Two characteristics of cancer cells that distinguish them from normal cells are their ability to produce elevated levels of ROS and their increased dependence on the antioxidant defense system^55^. The antioxidant system, which is considered an ideal target for cancer treatment, is important for maintaining redox balance. The UPS is a critical part of the antioxidant system because of its function in selectively degrading oxidatively damaged proteins^16^. In addition, antioxidant enzymes also play vital roles in quenching or metabolizing ROS^56^. In this study, we show that TRIB2 and PCBP2 modulate PMSB5 activity to reduce K48-ubiquitination of antioxidant enzyme GPX4 suggesting, that the UPS plays an additional role in preventing peroxidation other than targeted protein degradation. Given that TRIB2 is an antioxidant in liver cancer cells, TRIB2 might be an ideal target for the treatment of liver cancer.

In the present study, we also revealed that GPX4 is coordinately regulated by the three PSMB proteins in the proteasome. PSMB5 is stimulated by TRIB2 to degrade excessive Ub, while PSMB1 and PSMB2 are ineffective in degrading GPX4 because it is less ubiquitinated. On this basis, stimulation of PSMB5 by TRIB2 is a prerequisite for maintaining GPX4 stability. This finding also explains why the absence of TRIB2 facilitates the degradation of GPX4. We also found that GPX4 is more sensitive to degradation when Ub is abundantly available; however, the mechanism is still unclear and should be explained in the future.

In conclusion, we’ve established a model emerges in which TRIB2 cooperates with and stimulates PCBP2 to reduce the global K48-Ub level in liver cancer cells. We also demonstrated a novel pathway by which TRIB2 regulates the UPS in an E3-independent manner. Hence, we provide new evidence that the UPS is tightly regulated by TRIB2, and that targeting it might be a promising method for augmenting cell death under OS conditions.

## Supplementary information

Supplemental figure S1

Supplemental figure S2

Supplemental figure S3

Supplemental figure S4

Supplemental figure S5

Supplemental figure S6

Supplemental figure S7

Supplemental Legends

Supplemental table
