# Supplementary figures and images for: TRIB2 modulates proteasome function to reduce ubiquitin stability and protect liver cancer cells against oxidative stress
Source: Cell Death Dis. 2021 Jan 7;12(1):42. doi: 10.1038/s41419-020-03299-8 (PMC7791120; doi:10.1038/s41419-020-03299-8)

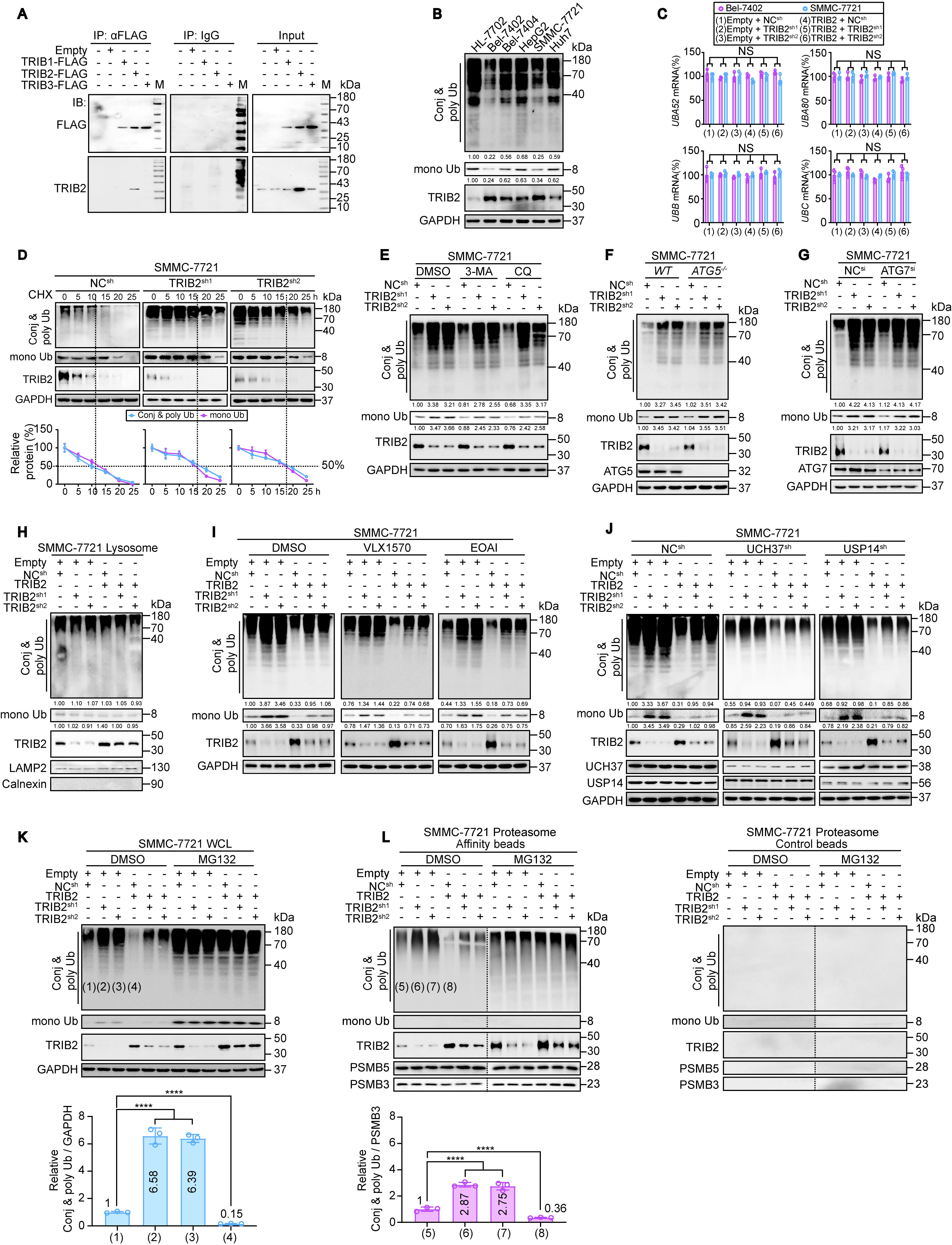

Supplement: Supplementary file 1 — Supplemental figure S1 [file 41419_2020_3299_MOESM1_ESM.tif]

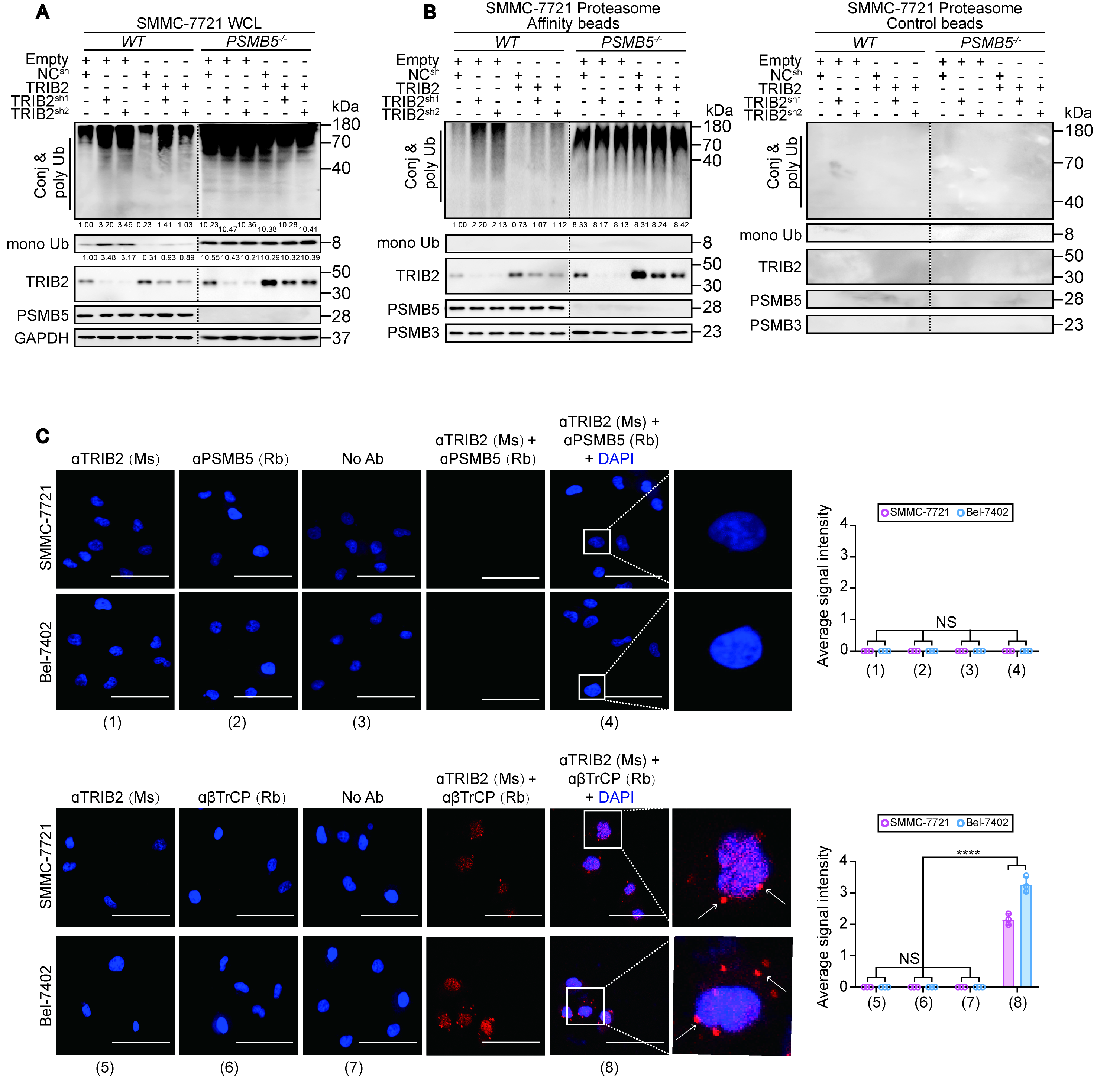

Supplement: Supplementary file 2 — Supplemental figure S2 [file 41419_2020_3299_MOESM2_ESM.tif]

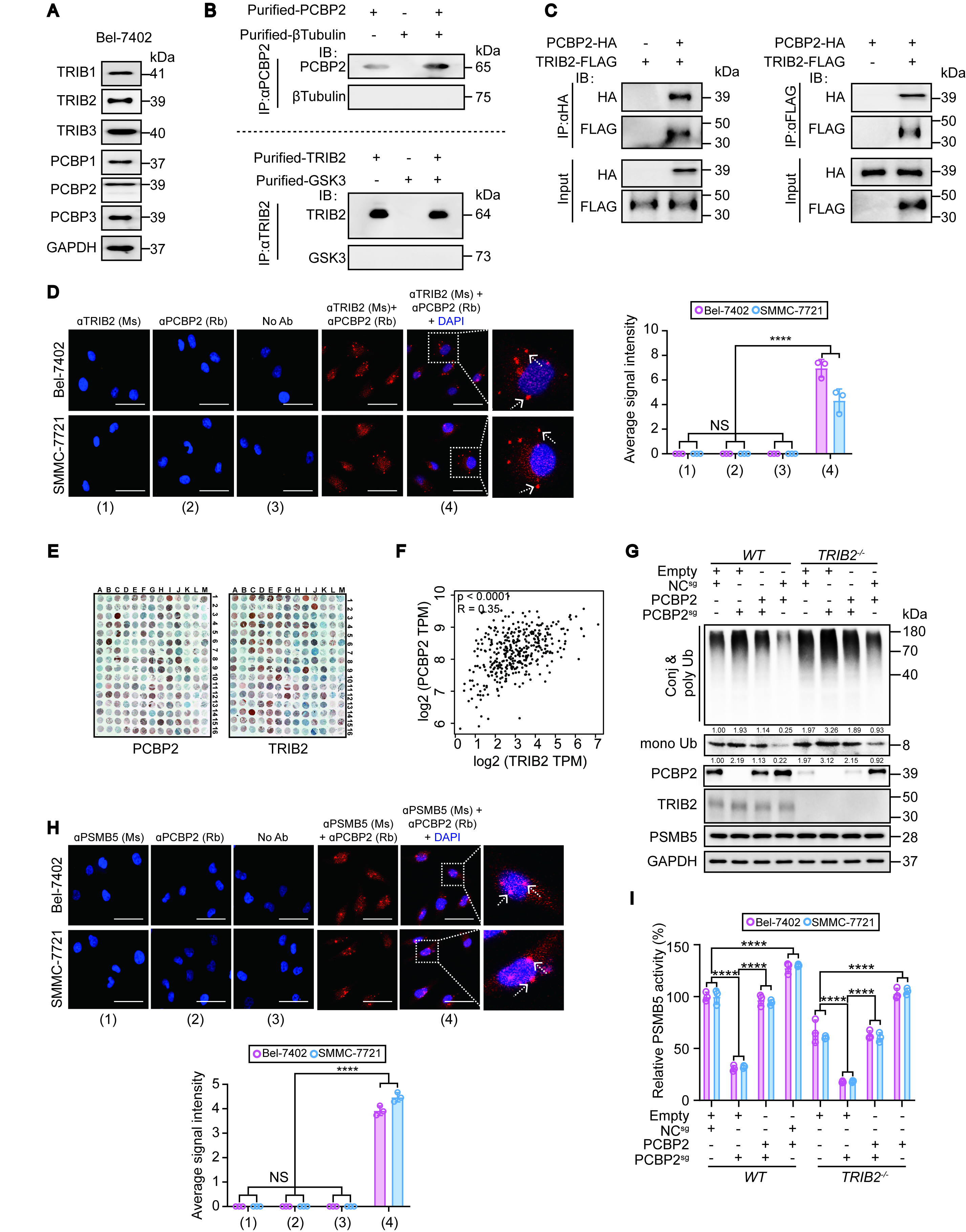

Supplement: Supplementary file 3 — Supplemental figure S3 [file 41419_2020_3299_MOESM3_ESM.tif]

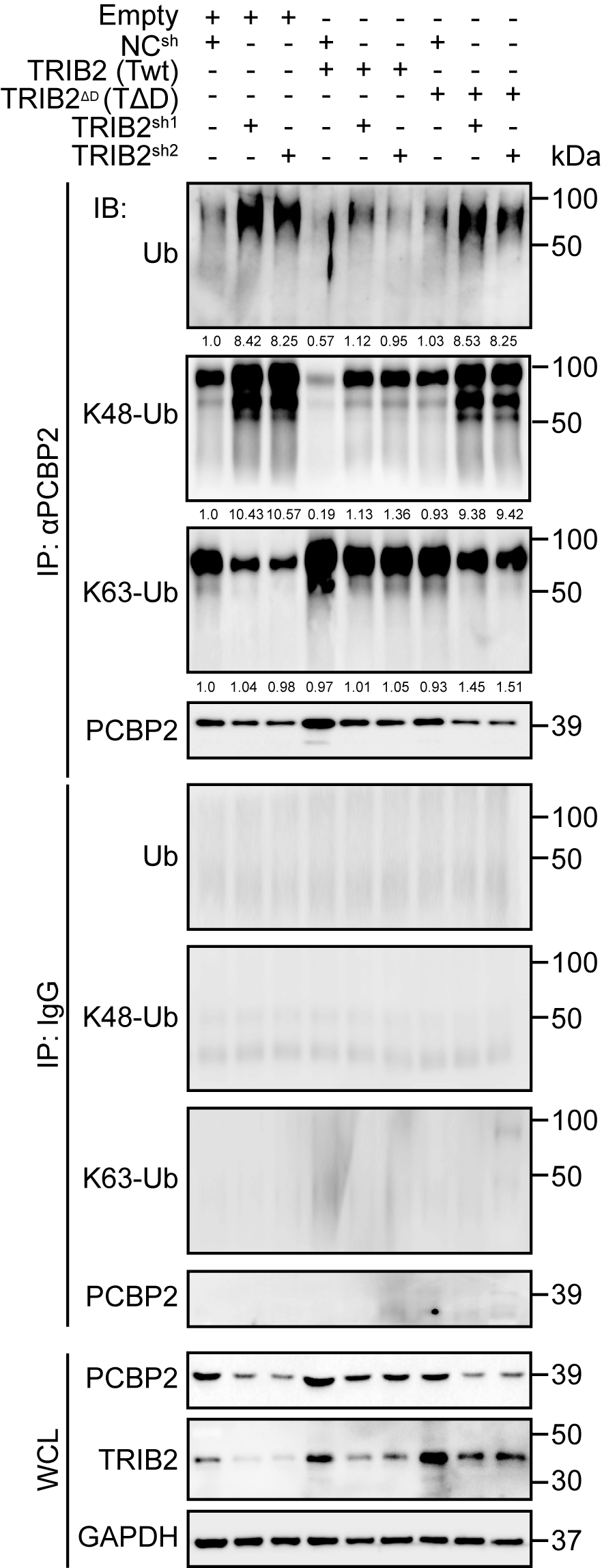

Supplement: Supplementary file 4 — Supplemental figure S4 [file 41419_2020_3299_MOESM4_ESM.tif]

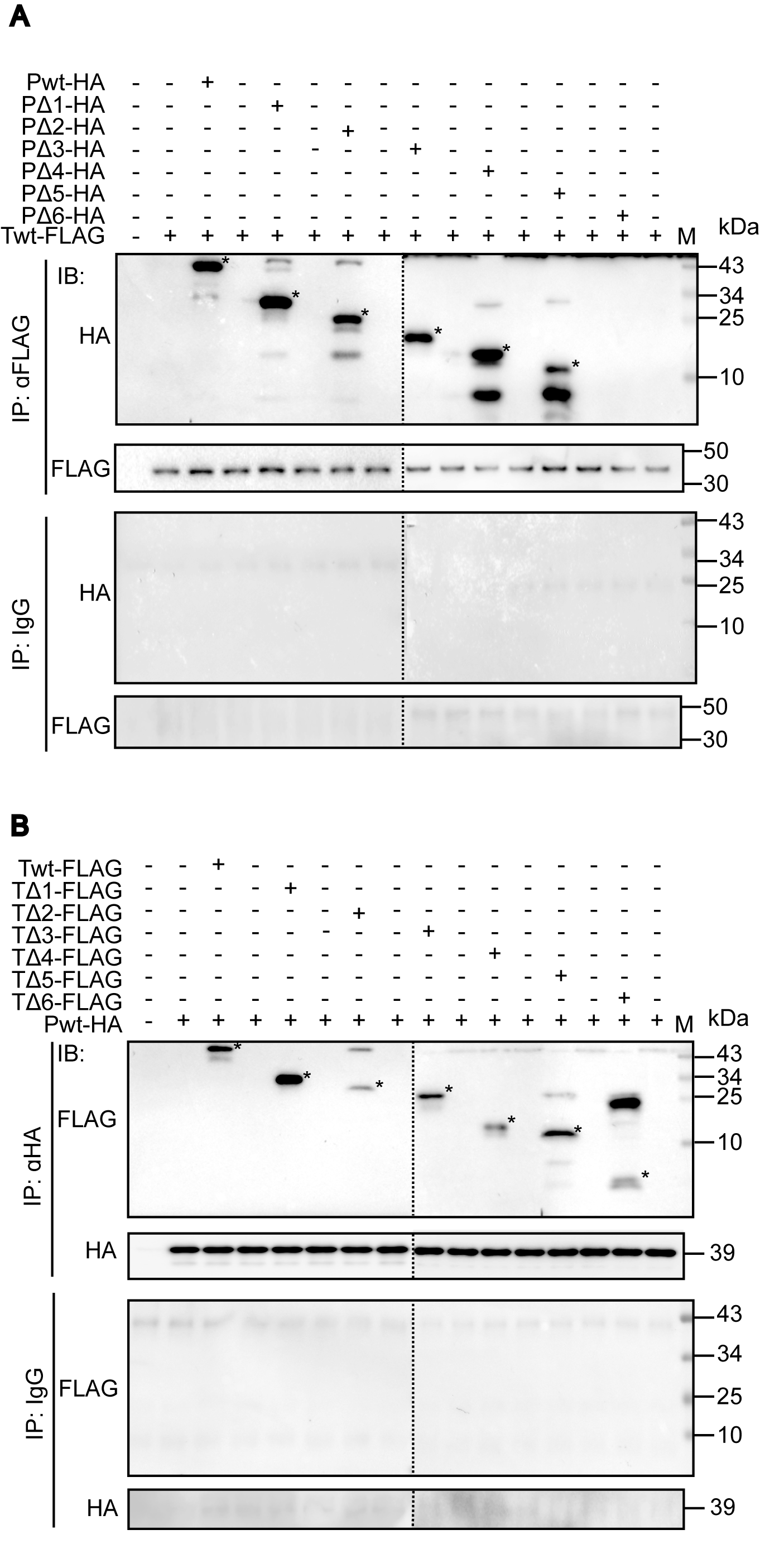

Supplement: Supplementary file 5 — Supplemental figure S5 [file 41419_2020_3299_MOESM5_ESM.tif]

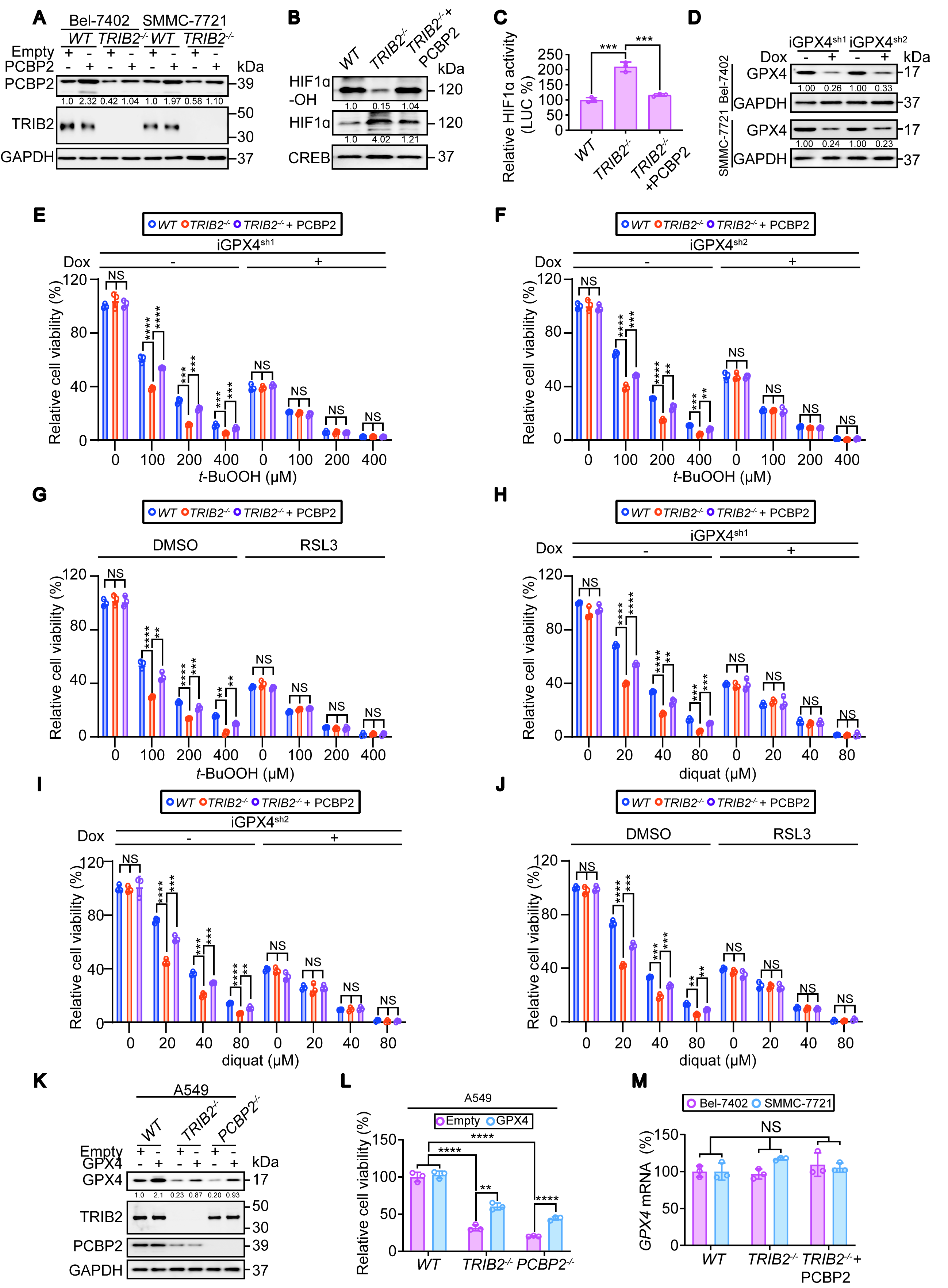

Supplement: Supplementary file 6 — Supplemental figure S6 [file 41419_2020_3299_MOESM6_ESM.tif]

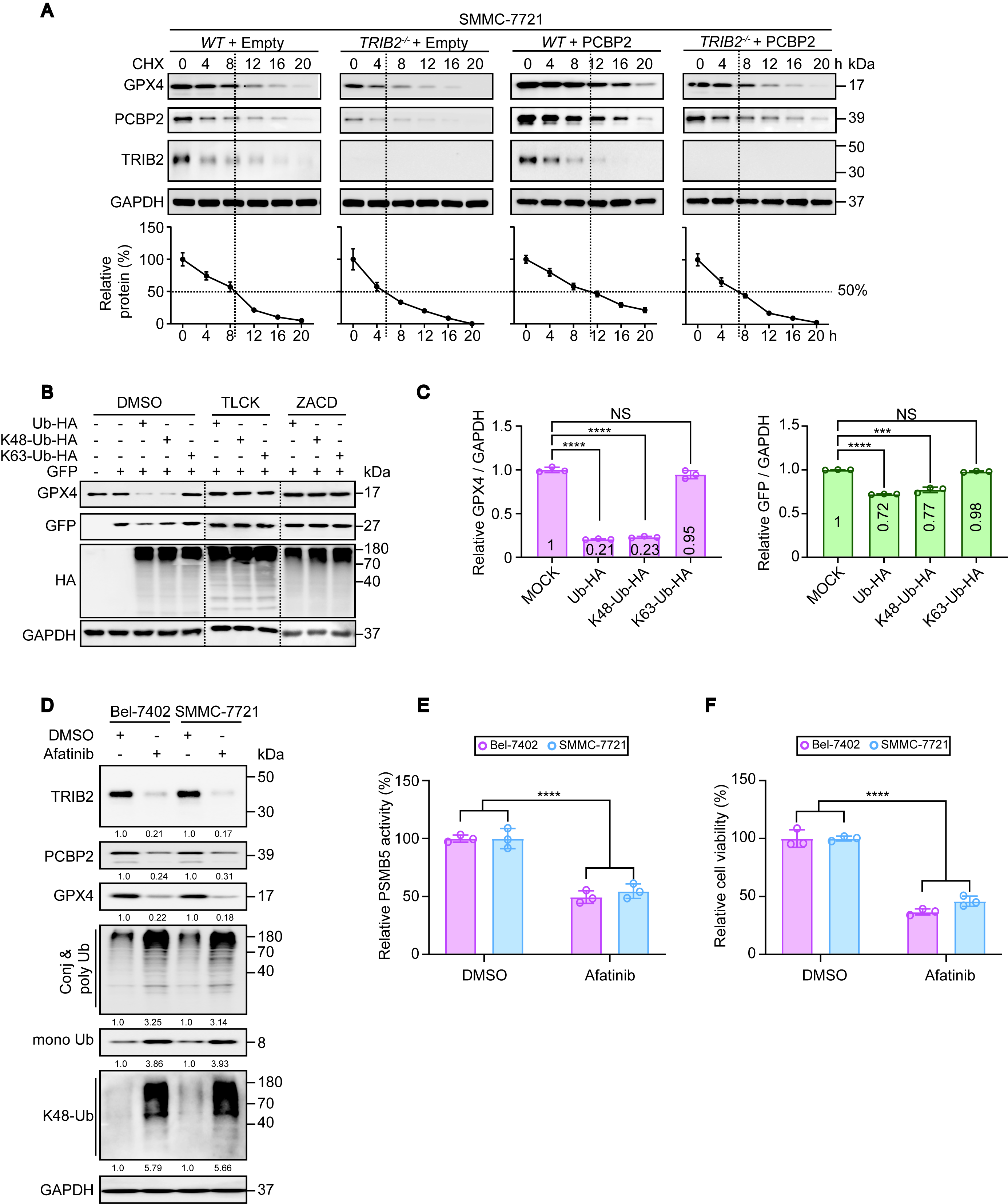

Supplement: Supplementary file 7 — Supplemental figure S7 [file 41419_2020_3299_MOESM7_ESM.tif]
