# Supplementary material for: TRIB2 modulates proteasome function to reduce ubiquitin stability and protect liver cancer cells against oxidative stress
Source: Cell Death Dis. 2021 Jan 7;12(1):42. doi: 10.1038/s41419-020-03299-8 (PMC7791120; doi:10.1038/s41419-020-03299-8)
Supplement: Supplementary file 8 — Supplemental Legends [file 41419_2020_3299_MOESM8_ESM.docx]

**Supplemental Figure and Table legends**

**Supplemental Figure legends**

**Fig. S1 TRIB2 regulates Ub levels via the proteasome**

**a** Specificity of the anti-TRIB2 antibodies. The exogenous FLAG-tagged TRIB1, TRIB2 and TRIB3 were ectopically expressed in HEK-293T cells, and immunoprecipated by anti-FLAG antibodies. TRIB2 was measured by IB using anti-TRIB2 antibodies (ab117981, Abcam), M, protein ladder. **b** Representative Immunoblot images of Ub in the established cell lines, as indicated. **c** mRNA levels of the *Ub* host gene in the Bel-7402 and SMMC-7721 cells with TRIB2 knocked down or overexpressed, as measured by qRT-PCR (n=3). Data were analyzed by one-way ANOVA and expressed as mean ± SD. NS, non-significance. **d** CHX chase experiments showed a significantly increased half-life of Ub (both conj & poly Ub and mono Ub) in the SMMC-7721 cells with TRIB2 knocked down. The relative protein levels of Ub were normalized to those of GAPDH, and the “0 h” point was arbitrarily set to 100%. Data were expressed as mean ± SD. **e-g** Ub in the SMMC-7721 cells with or without TRIB2 knocked down and treated with DMSO, 3-MA (5 mM, 24 h) or CQ (20 µM, 24 h) (**e**), and with ATG5 knocked out (**f**) or ATG7 knocked down (**g**). **h** TRIB2 had no effect on the Ub levels within lysosomes. Ub in the lysosomes was isolated from the same cells as shown in panel (**c**). The relative protein levels of conj & poly Ub and mono Ub were normalized to those of LAMP2 as calculated by ImageJ software and indicated just below the blots. **i-j** TRIB2 did not regulate Ub levels through DUBs. The SMMC-7721 cells as shown in panel (**c**) were treated with DMSO, VLX1570 (20 µM, 24 h) or EOAI (600 nM, 24 h) (**i**) or infected with shRNA targeting UCH37 or USP14 (**j**). **k-l** TRIB2 regulated Ub interaction with the proteasome. Ub at WCL (**k**) and in the proteasome (**l**) was measured in the same SMMC-7721 cells shown in panel (**c**) after treatment with DMSO or MG132 (8 µM, 12 h). The samples in panel (**l**) derived from the same experiment using control beads had been processed in parallel. The levels of conj & poly Ub were normalized to that of GAPDH at WCL (**k**), while normalized to that of PSMB3 in the isolated proteasome (**l**). Data were analyzed by one-way ANOVA and expressed as mean ± SD. ****, *P* < 0.0001. Images of all the immunoblots are representative of 3 independent experiments. The relative protein levels of conj & poly Ub and mono Ub were normalized to those of GAPDH as calculated by ImageJ software and indicated just below the blots (**e-g**, **i**, **j**).

**Fig. S2 TRIB2 suppresses Ub via PSMB5 in proteasome**

**a,b** Ub at the WCL and in proteasomes isolated from the SMMC-7721 cells with TRIB2 knocked down or overexpressed, in the presence or absence of PSMB5. The samples derived from the same experiment using control beads had been processed in parallel. The levels of conj & poly Ub and mono Ub were normalized to that of GAPDH at WCL (**a**), and that of PSMB3 in the isolated proteasome (**b**). Images of all the immunoblots are representative of 3 independent experiments. **c** Representative PLA images of TRIB2 and PSMB5. The interaction between TRIB2 and βTrCP is also shown in parallel (n=3); scale bar, 25 µm. The average PLA signals per cell were graphed beside the images. Data were analyzed by one-way ANOVA and expressed as mean ± SD. ****, *P* < 0.0001, NS, non-significance.

**Fig. S3 PCBP2 is essential for TRIB2 regulation of PSMB5 activity**

**a** Verification of the antibodies as indicated in Bel-7402 cells. **b** In vitro co-IP between purified PCBP2 and βTubulin and between purified TRIB2 and GSK3. **c** Reciprocal co-IP experiments in HEK-293T cells expressing exogenous PCBP2-HA or TRIB2-FLAG, as evaluated by anti-HA and anti-FLAG antibodies. **d** Direct interaction between endogenous PCBP2 and TRIB2 in the Bel-7402 and SMMC-7721 cells, as measured by a PLA experiment using the antibodies indicated (n=3); scale bar, 25 µm. The average PLA signals per cell were graphed beside images. Data were analyzed by one-way ANOVA and expressed as mean ± SD. ****, *P* < 0.0001; NS, non-significance**.** **e** TMA images performed by IHC using anti-PCBP2 and anti-TRIB2 antibodies (n=208). **f** Significant and positive correlation for TRIB2 and PCBP2 was obtained from the GEPIA tool (<http://gepia.cancer-pku.cn/>). **g** PCBP2 regulates Ub levels but is not upstream of TRIB2. Ub levels were measured in the control and Bel-7402 cells with or without TRIB2 and PCBP2 knocked out or overexpressed. The relative levels of conj & poly Ub and mono Ub were normalized to that of GAPDH and indicated below the blots. **h** Direct interaction between endogenous PCBP2 and PSMB5 in the Bel-7402 and SMMC-7721 cells, as measured by PLA experiment using the antibodies indicated (n=3); scale bar, 25 µm. The average PLA signals per cell were graphed below the images. Data were analyzed by one-way ANOVA and expressed as mean ± SD. ****, *P* < 0.0001; NS, non-significance**. i** PCBP2 isn’t the upstream of TRIB2 to regulate PSMB5 activity. PSMB5 activity was measured by a proteasome activity assay kit (AAT Bioquest) in Bel-7402 and SMMC-7721 cells under the same treatment as indicated in panel (**g**). (n=3) Data were analyzed by one-way ANOVA and expressed as mean ± SD. ****, *P* < 0.0001. Images of all the immunoblots are representative of 3 independent experiments.

**Fig. S4 The DQLVPD element of TRIB2 is essential for suppressing ubiquitination of PCBP2**

Total-, K48- and K63-ubiquitination of PCBP2, as immunoprecipitated by the anti-PCBP2 antibodies in SMMC-7721 cells with TRIB2 knocked down or overexpressed (either TRIB2 (Twt) or TRIB2^ΔD^ (TΔD)), and measured by the anti-K48, anti-K63, and anti-total-Ub antibodies. The relative levels of total-, K48- and K63-ubiquitination of PCBP2 were normalized to that of PCBP2 in the PCBP2-IPs, as calculated by ImageJ and indicated just below the blots. Images of immunoblots are representative of 3 independent experiments.

**Fig. S5 Binding between TRIB2 and PCBP2**

**a,b** co-IP were performed between TRIB2 and PCBP2 and their mutants in HEK-293T cells. Immunoprecipitation was performed by anti-FLAG **(a)** and anti-HA **(b)** antibodies and measured by IB using anti-HA and anti-FLAG antibodies. The asterisks indicate the specific bands that represent truncated versions of TRIB2 and PCBP2. M, protein ladder. Images of immunoblots are representative of 3 independent experiments.

**Fig. S6 PCBP2 and TRIB2 maintain cell viability via GPX4 under OS**

**a** PCBP2 in Bel-7402 and SMMC-7721 cells with or without TRIB2 knockout, in the presence or absence of PCBP2 overexpressing. **b** TRIB2 induced hydroxylation of HIF1α via PCBP2. Control and Bel-7402 cells with TRIB2 knocked out, in the presence or absence of PCBP2 overexpression were treated with MG132 (50 μM) for 6 h followed by DFO (25 μM) for 2 h before nuclear extracts were examined by IB using anti-HIF1α-OH, anti-HIF1α, and anti-CREB antibodies. The relative levels of HIF1α-OH and total-HIF1α were normalized to that of CREB and indicated below the blots. **c** TRIB2 reduced HIF1α activity via PCBP2. HIF1α luciferase reporter plasmids were co-transfected with Renilla luciferase reporter plasmids in control and Bel-7402 cells with TRIB2 knocked out, in the presence or absence of PCBP2 overexpression. Dual-luciferase activities were measured by a dual-luciferase reagent from Promega. Data were analyzed by one-way ANOVA and expressed as mean ± SD. ***, *P* < 0.001. **d** GPX4 in the Bel-7402 and SMMC-7721 cells with Dox-inducible shRNA targeting GPX4 in the presence or absence of Dox (700 ng/ml, 24 h). The relative levels of GPX4 were normalized to that of GAPDH and indicated below the blots. **e**-**j** Inhibition of GPX4 diminished the role of TRIB2 and PCBP2 in protecting SMMC-7721 cells against OS. SMMC-7721 cells transfected with Dox-inducible shRNAs targeting GPX4 (iGPX4^sh1^, **e, h**; iGPX4^sh2^, **f, i**) were pretreated with or without Dox (700 ng/ml, 24 h) (**e, f, h, i**). SMMC-7721 cells were also pretreated with or without RSL3 (5 µM) for 5 h (**g, j**). Then, the cells were exposed to the indicated concentration of *t*-BuOOH (**e**-**g**) or diquat (**h**-**j**) for another 24 h. Cell viability was determined based on neutral red staining, as described in the Materials and Methods section. Data were analyzed by one-way ANOVA and expressed as mean ± SD. **, *P* < 0.01; ***, *P* < 0.001; ****, *P* < 0.0001; NS, non-significance. **k** GPX4 in control and A549 cells with or without TRIB2 or PCBP2 knocked out in the presence or absence of overexpressed GPX4 (n=3). **l** Cell viability in the same cells as shown in panel (**k**) (n=3). Data were analyzed by one-way ANOVA and expressed as mean ± SD. **, *P* < 0.01; ****, *P* < 0.0001. **m** mRNA levels of *GPX4* in the Bel-7402 and SMMC-7721 cells under different treatments, as indicated (n=3). Data were analyzed by one-way ANOVA. NS, non-significance. Images of all the immunoblots are representative of 3 independent experiments.

**Fig. S7 Further verification for the interaction between GPX4 and TRIB2**

**a** CHX chase experiments of GPX4 in the control and SMMC-7721 cells with TRIB2 knocked out, with or without the simultaneous overexpression of PCBP2. The relative protein levels of GPX4 were normalized to those of GAPDH, and the “0 h” point was arbitrarily set to 100%. Data were expressed as mean ± SD. **b,c** Overexpressing Ub reduced GPX4 and exogenous GFP. Indicated Ub and GFP were ectopically expressed in Bel-7402 cells treated with or without TLCK (50 μM, 24 h) or ZACD (100 μM, 24 h). Representative IB images are shown in panel (**b**), while the relative protein expression of GPX4 and exogenous GFP were normalized to that of GAPDH and graphed in panel (**c**). Data were analyzed by Student’s *t*-test and expressed as mean ± SD. ****, *P* < 0.0001; NS, non-significance. **d** The effects of afatinib on TRIB2, PCBP2 and Ub. Bel-7402 and SMMC-7721 cells were treated with or without afatinib (10 μM) for 20 h before harvest for examination. The relative protein levels of TRIB2, PCBP2, GPX4, conj & poly Ub and mono Ub were normalized to that of GAPDH and the data are shown below the blots. **e** Afatinib reduced PSMB5 activity in Bel-7402 and SMMC-7721 cells. **f** Afatinib reduced cell viability, as measured by a neutral red staining-based method in Bel-7402 and SMMC-7721 cells following treating with Afatinib (10 μM) for 20 h. Data were analyzed by one-way ANOVA and expressed as mean ± SD. ***, *P* < 0.001; ****, *P* < 0.0001 (**e**, **f**). Images of all the immunoblots are representative of 3 independent experiments.

**Supplemental Table legends**

**Table S1.** siRNA sequences for target genes.

**Table S2.** Primers used for construction of plasmids

**Table S3.** Primers for qPCR
