# Supplementary material for: TRIB2 modulates proteasome function to reduce ubiquitin stability and protect liver cancer cells against oxidative stress
Source: Cell Death Dis. 2021 Jan 7;12(1):42. doi: 10.1038/s41419-020-03299-8 (PMC7791120; doi:10.1038/s41419-020-03299-8)
Supplement: Supplementary file 9 — Supplemental table [file 41419_2020_3299_MOESM9_ESM.doc]

**Supplementary Table S1-3**

**Table S1**

| **Name** | **5'-3'** |
| --- | --- |
| siATG7 | CCAACACACUCGAGUCUUUTT |
| siPOMP | GGACAGUAUUCCAGUUACUTT |
| siMYPT1 | GCAGCUCCUGUAGCUGUUATT |
| siGRP78 | GGUGGGCAAACAAAGACAUTT |

**Table S1. siRNA sequences for target genes.**

**Table S2Table S2. Primers used for construction of plasmids**

| **Name** | **5'-3'** |
| --- | --- |
| TRIB2-sg-F (lentiCRISPR v2) | CACCGGTTGTCGTCTATAAGGTCCG |
| TRIB2-sg-R (lentiCRISPR v2) | AAACCGGACCTTATAGACGACAACC |
| PCBP2-sg-F (lentiCRISPR v2) | CACCGGTTGAGAGAGGTTGATGCTG |
| PCBP2-sg-R (lentiCRISPR v2) | AAACCAGCATCAACCTCTCTCAACC |
| GPX4-sh1-F (pLKO) | CCGGGTGAGGCAAGACCGAAGTAAACTCGAGTTTACTTCGGTCTTGCCTCACTTTTTG |
| GPX4-sh1-R (pLKO) | AATTCAAAAAGTGAGGCAAGACCGAAGTAAACTCGAGTTTACTTCGGTCTTGCCTCAC |
| GPX4-sh2-F (pLKO) | CCGGGCACATGGTTAACCTGGACAACTCGAGTTGTCCAG GTTAACCATGTGCTTTTTG |
| GPX4-sh2-R (pLKO) | AATTCAAAAAGCACATGGTTAACCTGGACAACTCGAGTTG TCCAGGTTAACCATGTGC |
| Twt-F (pcDNA3.1) | GCGCGGATCCATGAACATACACAGGTCTACCCCC |
| TΔ1-F (pcDNA3.1) | GCGCGGATCCATGACTCCGAACTTGTCGCATTGCGTT |
| TΔ2-F (pcDNA3.1) | GCGCGGATCCATGCTGGCACCGTGCTTTTGCCTGTCT |
| TΔ3-F (pcDNA3.1) | GCGCGGATCCATGGAGGCAGCCAGACTGTTCTACCAG |
| TΔ4-F (pcDNA3.1) | GCGCGGATCCATGATTCTGCGGGGAGATGATGATTCC |
| TΔ5-F (pcDNA3.1) | GCGCGGATCCATGCGGTACCCTTTCCATGACATTGAA |
| TΔ6-F (pcDNA3.1) | GCGCGGATCCATGGAAATTCTGGACCATCCTTGGTTT |
| Twt-R (pcDNA3.1) | GCGCCTCGAGTCACTTGTCATCGTCGTCCTTGTAATCTCAGTTAA AGAAAGGGTCCAAGT |
| TΔD-R1 (pcDNA3.1) | CTTCCATGTTGACAGACACTTCCTTAGCACCATA |
| TΔD-F2 (pcDNA3.1) | GTCAACATGGAAGAGAACTTG |
| TΔH-R1 (pcDNA3.1) | GCTAAAATCTGTAGAGTCCAGAATTTCCTGCGAGG |
| TΔH-F2 (pcDNA3.1) | TCTACAGATTTTAGCGTCTCGAATTC |
| Pwt-F (pcDNA3.1) | GCGCGGATCCATGGACACCGGTGTGATTGAAGGTGG |
| PΔ1-F (pcDNA3.1) | GCGCGGATCCATGGAAGGGAATTGTCCTGAGAGAATTAT |
| PΔ2-F (pcDNA3.1) | GCGCGGATCCATGAGGCTGGTGGTCCCTGCTAGTCAGT |
| PΔ3-F (pcDNA3.1) | GCGCGGATCCATGATTCCACAATCCATCATTGAGTGTG |
| PΔ4-F (pcDNA3.1) | GCGCGGATCCATGAGCACAGGCAGCGACAGTGCGAGCT |
| PΔ5-F (pcDNA3.1) | GCGCGGATCCATGATGCAACAGTCTCATTTTCCCATGAC |
| PΔ6-F (pcDNA3.1) | GCGCGGATCCATGTTTGATTGGCTGCATAATCGGGCGTC |
| Pwt-R (pcDNA3.1) | GCGCCTCGAGCTAAGCGTAGTCTGGGACGTCGTATGGGTACTAGC TGCTCCCCATGCCACCCG |
| PΔK-R (pcDNA3.1) | GCGCCTCGAGCTAAGCGTAGTCTGGGACGTCGTATGGGTACTAGCTGCTCCCCATGCCACCCGTCTCCGAAGTCTGAGCAGATGCAT |

**Table S3. Primers for qPCR**

| **Table S3** | |
| --- | --- |
| **Name** | **5'-3'** |
| UBA52-F | AAGACAAGGAGGGTATCCCAC |
| UBA52-R | TGTTGTAGTCTGAGAGAGTGCG |
| UBA80-F | CTGGAAGATGGACGTACTTTGTC |
| UBA80-R | CGACGAAGGCGACTAATTTTGC |
| UBB-F | GGTCCTGCGTCTGAGAGGT |
| UBB-R | GCCTTCACATTTTCGATGGTGT |
| UBC-F | CTGGAAGATGGTCGTACCCTG |
| UBC-R | GGTCTTGCCAGTGAGTGTCT |
| PCBP2-F | CTTTGGCTGGACCCACTAATG |
| PCBP2-R | CCCTGTACTCTCTCGTATTTCCT |
| GPX4-F | GAGGCAAGACCGAAGTAAACTAC |
| GPX4-R | CCGAACTGGTTACACGGGAA |
